# Brain‐Adhesive Bioelectronics With Shape‐Morphable and Biodegradable Properties for Stable Brain Signal Monitoring

**DOI:** 10.1002/advs.202518255

**Published:** 2026-03-03

**Authors:** Heewon Choi, Soeun Kim, Sumin Kim, Jaehyun Park, Soojung An, Sungjun Yoon, Mikyung Shin, Sangho Cho, Donghee Son

**Affiliations:** ^1^ Center for Neuroscience Imaging Research Institute For Basic Science Suwon Republic of Korea; ^2^ Department of Electrical and Computer Engineering Sungkyunkwan University Suwon Republic of Korea; ^3^ Extreme Materials Research Center Korea Institute of Science and Technology Seoul Republic of Korea; ^4^ Department of Intelligent Precision Healthcare Convergence Sungkyunkwan University Suwon Republic of Korea; ^5^ Department of Chemical and Biomolecular Engineering Yonsei University Seoul Republic of Korea; ^6^ Department of Biomedical Engineering Sungkyunkwan University Suwon Republic of Korea; ^7^ Nanoscience and Technology KIST School Korea University of Science and Technology Seoul Republic of Korea

**Keywords:** biodegradable self‐healing polymer, electrocorticography, magnetic resonance imaging compatibility, neural interface, tissue adhesion, transient electronics

## Abstract

Accurate and temporary monitoring of brain activity is essential for diagnosing and treating neurological diseases. Conventional nondegradable electrocorticogram (ECoG) devices require removal surgery, thus increasing the risk of infection and tissue damage. Moreover, existing devices typically fail to conform to soft, dynamic brain tissue, thus resulting in unsatisfactory adhesion, signal loss, and mechanical mismatch. Herein, we present the development of a brain‐adhesive sensor (B‐Sensor) with shape‐morphable and biodegradable characteristics, enabling stable ECoG signal monitoring within a clinically feasible window for patient application. The B‐Sensor is fabricated on a polyurethane elastomer incorporated with polycarbonate, which possesses a low glass transition temperature and dynamic bonding, thereby providing biodegradability, stretchability, stress relaxation, and even self‐healing capabilities. A tissue‐adhesive hydrogel in the B‐Sensor ensures conformal cortical adhesion, whereas ultrathin molybdenum electrodes in an open‐mesh layout maintain stable performance under cyclic strain and minimize magnetic resonance imaging (MRI) artifacts. The device degrades naturally under physiological conditions, retains impedance stability during use, and exhibits excellent cell viability. In vivo experiments show that the B‐Sensor reliably records baseline activity, somatosensory evoked potentials, and 4‐aminopyridine‐induced epileptiform discharges with high precision. This study demonstrates a bioresorbable, tissue‐adhesive ECoG platform that enables safe, artifact‐free monitoring of both normal and pathological brain activity, thus offering a new design paradigm for next‐generation implantable bioelectronics.

## Graphical abstract

A brain‐adhesive sensor (B‐Sensor) was developed by integrating a self‐healing biodegradable elastomer, a tissue‐adhesive hydrogel, and molybdenum electrodes. The B‐Sensor adheres to brain tissue, conforms to cortical curvatures, and maintains stable electrical performance over the intended period for reliable recording of spatiotemporal brain activity while undergoing gradual degradation.

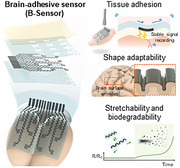

## 1 Introduction

Many clinical situations require temporary monitoring of brain activity during the diagnosis and treatment of neurological diseases. A representative example is the necessity to accurately locate the lesion before epilepsy surgery [1, 2, 3] or to monitor disease progression and recovery in real time in acute conditions such as traumatic brain injury or meningitis [4, 5, 6]. In these cases, electrocorticogram (ECoG) sensors are used; however, they require surgical removal through a second craniotomy, which poses the risk of infection, bleeding, and tissue damage [7, 8]. As a result, concerns related to reoperation risk have limited the clinical use of ECoG largely to the acute stage monitoring. Therefore, the development of a biodegradable ECoG sensor [8, 9, 10, 11, 12] that naturally decomposes in the body after use would significantly improve patient safety and satisfy the clinical requirements [4, 5, 13, 14] for personalized neural signal monitoring. Additionally, imparting spontaneous degradability to existing bioelectronic platforms can reduce the surgical burden, shorten hospital stays, and enable the establishment of effective follow‐up treatment plans.

The cortical surface, which features complex curvatures ranging from a few millimeters to several millimeters, is affected easily by continuous micromotions driven by physiological factors such as breathing, heartbeat, and body movement [15, 16, 17, 18, 19]. To achieve a stable brain tissue–device interface under the dynamic and mechanically challenging in vivo environment, the brain sensor must exhibit spontaneous shape adaptability to the mechanical properties of the cortex tissue [20, 21, 22, 23]. The failure of the sensing electrode to achieve intimate contact with the cortex or exhibiting excessive stiffness can result in elevated noise levels, signal‐to‐noise ratio attenuation [24, 25, 26, 27], and local tissue damage [28, 29, 30, 31, 32]. In particular, for implantation without using conventional adhesives or sutures, stable positioning on the cortex is achievable only when the device possesses sufficient tissue adhesion [33, 34, 35, 36, 37]. Moreover, the sensors should maintain their intrinsic electrical function without malfunctioning under irregular deformation caused by repeated brain movements; this necessitates the appropriate stretchability for stable signal recording during the intended monitoring period. In addition to the prerequisites for reliable sensing performance, patients with neurological diseases typically require repeated magnetic resonance imaging (MRI) scans to monitor lesions and assess treatment responses in a non‐invasive manner. However, conventional brain sensors that utilize noble metals distort MRI images. Furthermore, conventional electrodes and interconnects can undergo heating or physical displacement under magnetic fields, thus limiting their clinical application. Therefore, metallic materials that do not interfere with the MRI operation must be selected, and the sensor design must be optimized to minimize MRI‐induced Joule heating [38, 39, 40].

**FIGURE 1:**
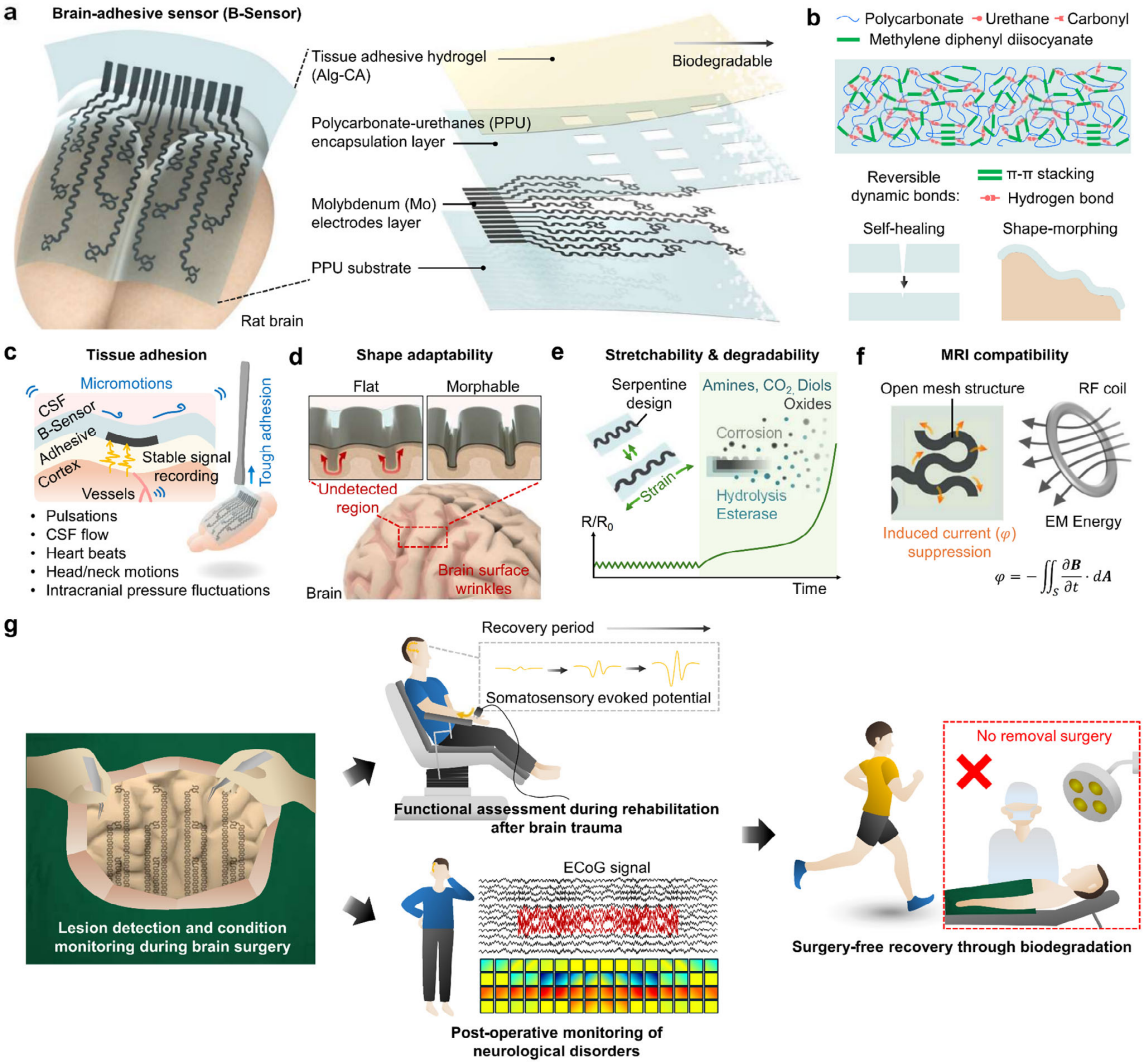
Schematic and concept of a brain‐adhesive sensor (B‐Sensor). (a) Overall B‐Sensor architecture comprising a PPU substrate, ultrathin molybdenum electrodes patterned in a serpentine structure, a PPU encapsulation layer, and a tissue‐adhesive hydrogel coating. Twelve electrodes are arranged to cover the cortical surface, including motor and visual areas. (b) Chemical structure and key features of the PPU elastomer, showing self‐healing and shape morphing capability through dynamic bonding. (c) Conceptual illustration of stable tissue adhesion, which preserves conformal contact under cortical micromotions. (d) The shape‐morphing property of the B‐Sensor, which adapts to brain surface curvatures for precise and stable signal acquisition. (e) Serpentine design of molybdenum electrodes provides electrical stability over the intended period and then gradually dissolves under physiological conditions. (f) MRI compatibility of the open‐mesh electrode design, which suppresses RF‐induced heating and minimizes imaging artifacts. (g) Conceptual clinical application of the B‐Sensor. The B‐Sensor enables precise lesion localization during surgery and supports reliable brain‐signal monitoring throughout the rehabilitation period. In cases of physical brain trauma, it allows longitudinal tracking of somatosensory evoked potential to evaluate prognosis. For neurological disorders, it can detect and map lesion sites or epileptogenic foci to guide therapy. Finally, it biodegrades in vivo after the intended monitoring window, eliminating the need for removal surgery.

In this study, we develop a 12‐channel brain‐adhesive sensor (B‐Sensor) with cortex‐shape‐morphable and biodegradable properties to monitor artifact‐free ECoG signals (Figure 1a). The B‐sensor comprises four layers: (i) a self‐healing and fully biodegradable substrate based on a polycarbonate‐based polyurethane (PPU) elastomer, (ii) an ultrathin molybdenum (Mo) electrode layer, (iii) a PPU encapsulation layer, and (iv) a tissue‐adhesive alginate‐conjugated catechol (Alg‐CA) hydrogel. The use of 12 electrodes is sufficient to provide complete coverage of both the motor and visual cortices. PPU with a low glass transition temperature (*T*_g_) and dynamic bonds can provide spontaneous self‐healing and dynamic stress‐adaptable properties (Figure 1b). To further stabilize the sensor–brain interface, an Alg‐CA hydrogel was coated on the device surface, thus allowing the electrode array to remain stably attached to the cortex. This adhesion minimized noise interference from continuous brain micromotions caused by pulsations, cerebrospinal fluid flows, heartbeats, postural changes, and intracranial pressure variations (Figure 1c). The robust tissue‐adhesion capability of the B‐sensor minimizes undesired shear stress on the brain while forming a stable interface.

In particular, whereas the ability to firmly adhere to the brain tissue is highly important, conformity to cortical wrinkles is critical. In this regard, PPU, which has low *T*_g_ and typical viscoelastic properties, allows for polymer backbone rearrangement under physiological temperature (i.e., 40°C) and is highly beneficial for achieving tissue‐adaptable performance. Thus, the PPU substrate enables intimate contact by undergoing self‐induced mechanical deformation in accordance with the cortical curvatures. Supported by capillary and van der Waals forces, these synergistic effects enable conformal interfacing with the complex brain surface, including the sulci, and precise signal acquisition (Figure 1d).

To achieve strain‐insensitive properties during in vivo implantation and repeated brain micromotions, the B‐sensor was designed with ultrathin molybdenum patterned in a serpentine open‐mesh layout. This geometry provided high elasticity and preserved the electrical performance under cyclic strain (10 % for 1000 cycles) (Figure 1e, left). Importantly, the B‐Sensor remained stable in vivo for approximately three weeks, after which it gradually degraded into molybdenum oxides, CO_2_, amines, and other small molecules that were naturally excreted (Figure 1e, right). Of course, although MRI compatibility does not imply neural signaling during MRI operation, the open‐mesh design of Mo electrodes can reduce RF‐induced currents and suppress heat generation (Figure 1f). Consequently, adopting the open‐mesh design minimizes MRI artifacts without tissue burning.

The B‐sensor was implanted on the cerebral cortex of rat, where stable contact was established, and neural activities were successfully recorded, including sensory signals evoked by tactile stimulation and those associated with epileptic seizures. Over time, the B‐sensor can be gradually degraded and disappear naturally in vivo. It enables clinically relevant, stable neural monitoring over extended time windows without imposing the burden of secondary surgery. Accordingly, ECoG‐based applications such as preoperative seizure monitoring can now be supported without the burden of secondary surgery. Furthermore, the reduced implantation burden enables additional clinical applications. In patients with neurological disorders such as epilepsy, the implanted B‐Sensor can capture seizure‐related brain activity in real time to guide surgical strategies, whereas in patients with physical brain trauma, it can evaluate functional recovery by recording somatosensory‐evoked responses during rehabilitation (Figure 1g) [1, 2, 3, 4, 5, 30]. Thus, this study highlights a materials–device strategy that ensures both patient safety and treatment efficiency through a continuous process involving implantation, neural signal recording, and natural biodegradation. A comparison of representative flexible and stretchable ECoG platforms that address these design considerations is summarized in Table S1.

## 2 Results and Discussion

### 2.1 Synthesis of the Biodegradable Self‐Healing Polymer

To impart biodegradability, self‐healing, and adhesive properties, we designed a polyurethane comparing two segments: a biodegradable polycarbonate diol (soft segment) and a rigid aromatic diisocyanate (hard segment) linked via urethane bonds. Self‐healing originates from the intrinsic viscoelasticity of polyurethanes and hydrogen bonding between N─H and C═O groups [41, 42]. Aliphatic polycarbonate diols were selected for their biocompatibility, biodegradability, and low in vivo toxicity, which render them promising candidates for biomedical applications [43, 44, 45, 46].

**FIGURE 2:**
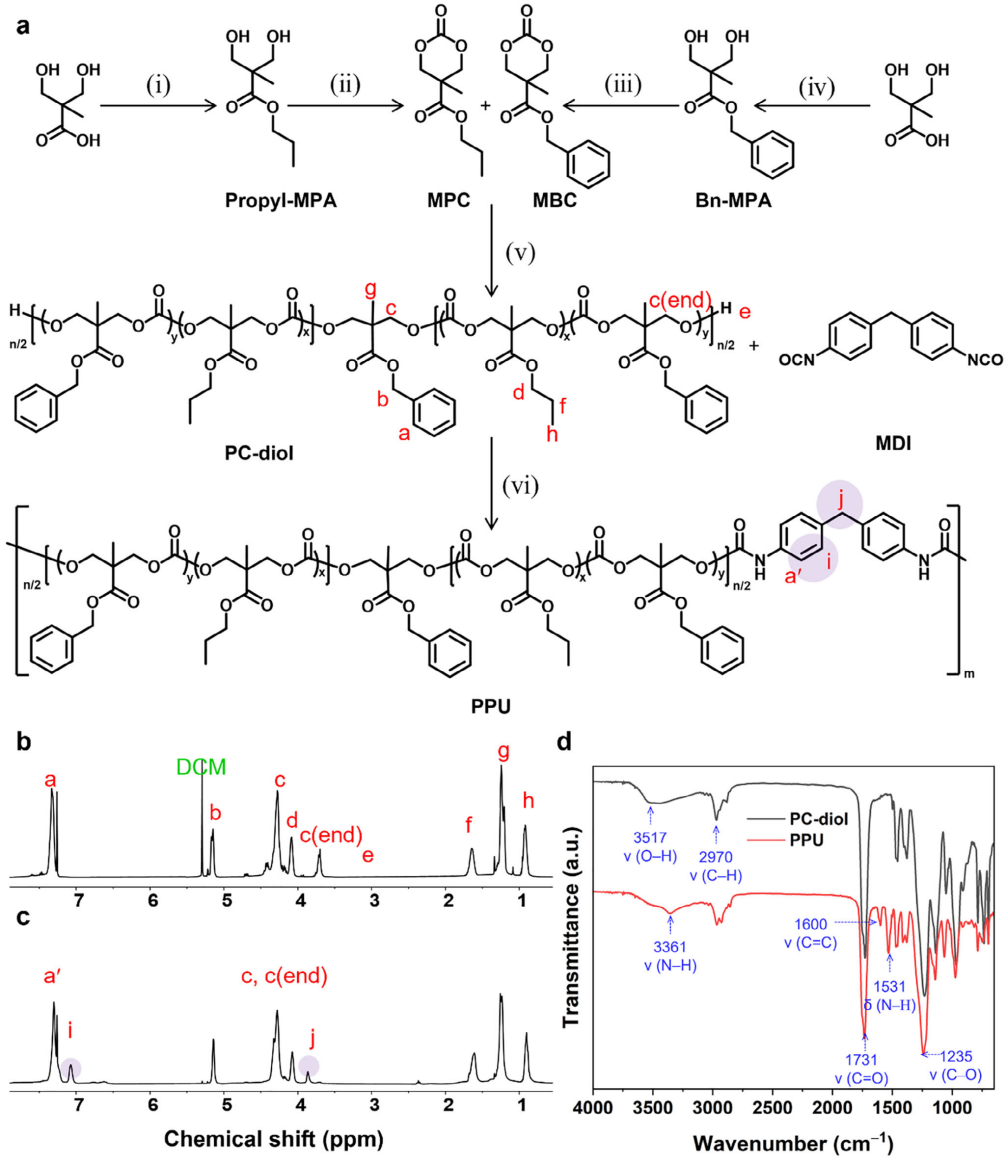
Synthesis of PPU. (a) Scheme for the synthesis of PPU: (i) propyl bromide, Et_3_N, MeCN, 80°C, overnight, (ii) ethyl chloroformate, Et_3_N, THF, 0°C to room temperature, overnight (iii) ethyl chloroformate, Et_3_N, THF, 0°C to room temperature, overnight (vi) benzyl bromide, KOH, DMF, 100°C, overnight. (v) Bn‐MPA, DBU, DCM, room temperature, 2 h (vi) DBTDL, DCM, room temperature, 24 h. (b,c) ^1^H NMR spectra (CDCl_3_, 400 MHz) of (b) PC‐diol and (c) PPU. (d) FTIR spectra of the reaction products: PC‐diol and PPU.

PPU was synthesized in three steps: (i–iv) preparation of cyclic carbonate monomers, (v) synthesis of polycarbonate diols, and (vi) polycondensation with diisocyanates (Figure 2a). Two monomers, i.e., 5‐methyl‐5‐propyloxycarbonyl‐1,3‐dioxane‐2‐one (MPC) and 5‐methyl‐5‐benzyloxycarbonyl‐1,3‐dioxane‐2‐one (MBC), were prepared via the esterification of 2,2‐bis(methylol)propionic acid (bis‐MPA) with alkyl halides, followed by cyclization with ethyl chloroformate, thus yielding functionalized six‐membered cyclic carbonates; propyl‐functionalized cyclic carbonate is MPC, whereas benzyl‐functionalized cyclic carbonate is MBC. The detailed synthesis procedures are described in the experimental and each chemical structure was confirmed by observing ^1^H/^13^C NMR spectra (Figures S1–S4). The monomers were purified via recrystallization or column chromatography to ensure controlled polymerization.

The ring‐opening polymerization of MPC and MBC was catalyzed by an organobase under moisture‐free conditions using benzyl 3‐hydroxy‐2‐(hydroxymethyl)‐2‐methylpropanoate (Bn‐MPA) as the initiator, which affords polycarbonate diol (PC‐diol) (Figure S5). The feed molar ratio of [Bn‐MPA]:[MPC]:[MBC] = 1:5:4 endowed PC‐diol with a final composition of 1:4:2.8 and M_n_ ≈ 1.8 kDa (≈ 80 % conversion). This was confirmed via ^1^H NMR spectroscopy, where the proton resonances were observed for each characteristic methylene peak of MPC (f) at 1.63 ppm, MBC (b) at 5.15 ppm, and methylene next to the hydroxyl end group (c end) at 3.7 ppm (Figure 2b).

Finally, PC‐diol was polycondensed with 4,4′‐methylenebis(phenyl isocyanate (MDI) using dibutyltin dilaurate (DBTDL) as a catalyst. MDI was selected to induce crystalline hard‐domain microphases via *π–π* intermolecular stacking. The appearance of new peaks at 7.08 ppm (Figure 2c‐i aromatic MDI) and 3.87 ppm (Figure 2c‐j methylene between benzene rings of the MDI), as well as the shifted methylene peak from 3.7 to 4.27 ppm (adjacent to the hydroxyl end group of PC‐diol), confirmed the successful urethane bond formation in the resulting PPU (Figure S6). The results of FT‐IR spectroscopy further supported urethane formation, by observing disappearance of broad O─H stretching (3517 cm^−1^) of PC‐diol and showing N─H stretching at 3361 cm^−1^_,_ N─H bending at 1531 cm^−1^ and characteristic carbonyl (C═O) bonds in urethane and carbonate bonds around 1750–1731 cm^−1^, including free (non‐H‐bonded) carbonyl groups of hard and soft segments and H‐bonded carbonyls between them were presented (Figure 2d) [47, 48, 49]. The disappearance of the MDI NCO peak at 2281 cm^−1^ indicated complete consumption of isocyanate groups (Figure 2d) [47, 48, 49]. The 1600 cm^−1^ peak assigned to aromatic C═C stretching of methylene diphenyl units confirmed the incorporation of MDI into PPU [49]. Additionally, an increase in the molecular weight from 2 to 12 kDa or from 5 to 13 kDa was confirmed through size‐exclusive chromatography (Figure S7). The equivalent stoichiometry between the diols and diisocyanate is critical. PPU_6 wt. %, a lower MDI content (< 6 wt. % for M_n_ of PC‐diol ≈ 5 kDa) yielded overly soft, rapid healing materials, whereas PPU_13 wt. %, a higher MDI content (> 13 wt. % for *M*_n, NMR_ of PC‐diol < 2 kDa) yielded stable, stretchable, and self‐healable films at room temperature (RT). PPU with <6 wt. % MDI exhibited a lower degradation temperature (*T*_d_) of 203°C, whereas a PPU with >13 wt. % of MDI increased the *T*_d_ of 233°C (Figure S8a). Similarly, a low MDI content yields a lower glass transition temperature (*T*_g_) of 7.8°C, whereas a higher MDI content raises *T*_g_ to 9.4°C and introduces an additional *T*_g_ at 66°C, thus reflecting the higher hard‐segment fraction (Figure S8b). Consequently, elastic, stretchable films with strong cohesion and self‐healing capability were formed. Thus, PPU was selected as the biodegradable polymer for constructing the B‐Sensor.

### 2.2 Tissue‐Conformable Interfacing

**FIGURE 3:**
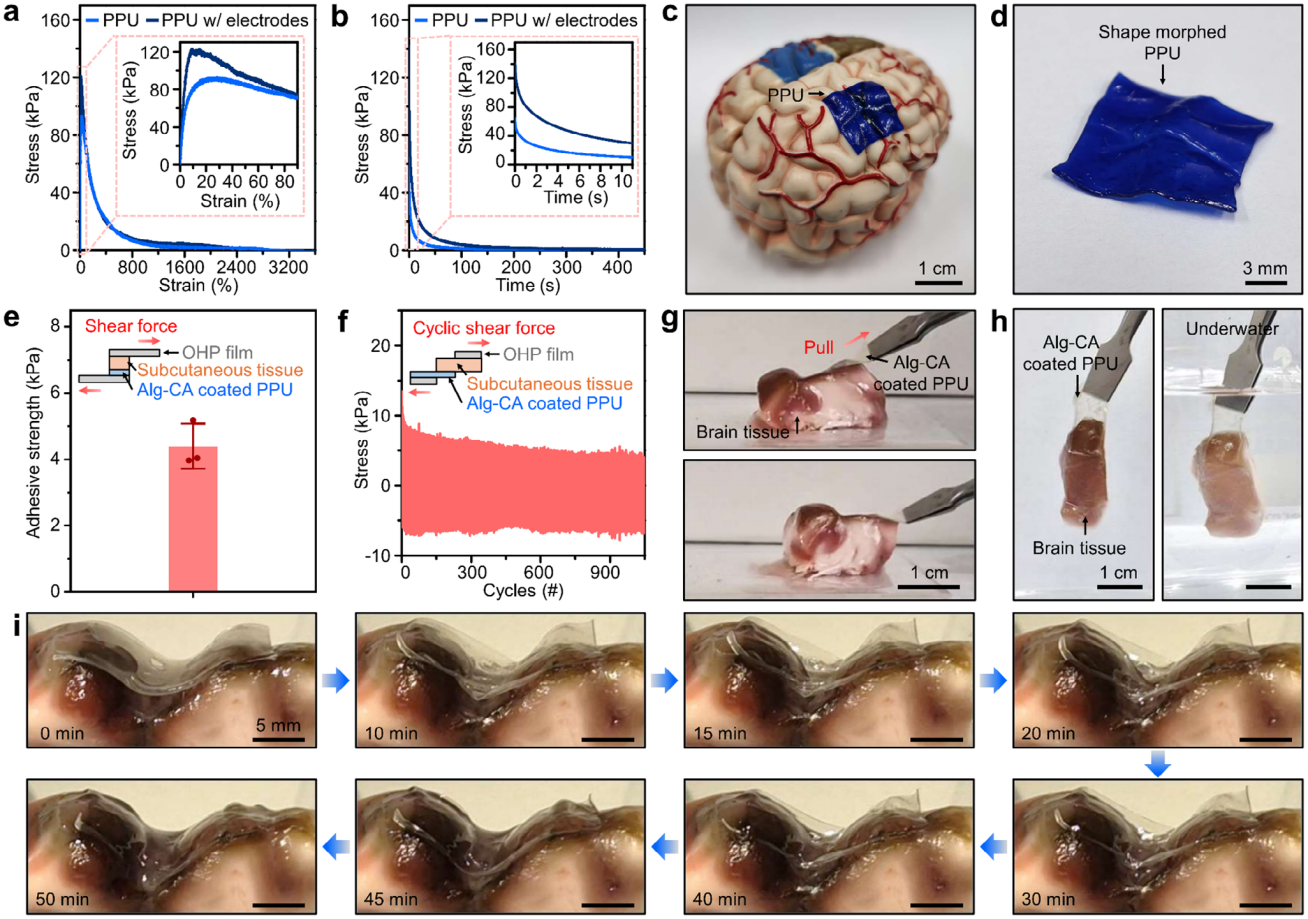
Mechanical and adhesive characteristics of the PPU substrate and adhesive hydrogel coating. (a) Stress–strain curve of PPU and PPU with molybdenum electrodes. Representative data were plotted among repeated tests (n = 3). (b) Stress relaxation behavior of PPU and PPU with molybdenum electrodes under 30 % strain. Representative data were plotted among repeated tests (n = 3). (c,d) Photographs showing conformal contact of the PPU on the brain model (c) and shape‐morphed PPU (d). (e) Shear adhesive strength of Alg‐CA‐coated PPU on rat subcutaneous tissue. The experiments were conducted in triplicate. Data were presented as mean ± SD. (f) Adhesive force under 1000 repeated cycles of 10 % strain. (g,h) Ex vivo bovine brain experiment demonstrating adhesion (g) and its retention underwater (h). (i) Shape‐morphing behavior of Alg‐CA‐coated PPU on the brain surface, showing gradual adaptation to cortical curvature.

The synthesized PPU polymer exhibited a Young's modulus of 1.4 MPa when deformed at a rate of 20 mm min^−1^ during tensile testing (Figure 3a). The stress–strain curve shows that the stress decreased after reaching its maximum at 10 % strain. The typical viscoelastic behavior is highly beneficial for adapting to the mechanical stiffness of living tissues. A dynamic mechanical analysis was also performed. After applying 30 % strain to the specimen and monitoring the stress change, 96 % of the initial stress was dissipated within 60 s (Figure 3b). This indicates that rapid rearrangement of the polymer chains and repeated breaking and reformation of dynamic bonds occurred. Since the *T*_g_ of the PPU polymer is low (i.e., 9.4°C), such rapid stress relaxation is possible, thus enabling the polymer to exhibit self‐healing properties (Figure S9). Furthermore, the PPU conformed to the complex geometry of the brain model when mounted (Figure 3c), and this conformity was also observed immediately after detachment (Figure 3d).

To impart tissue adhesion, the surface of the PPU was coated with an alginate‐based adhesive hydrogel functionalized with catechol groups (Alg‐CA). The tissue adhesion of Alg‐CA arises from the catechol functional groups, which can form multiple noncovalent interactions (i.e., hydrogen bonding, *π–π* stacking, and hydrophobic interactions) with functional groups present on wet biological tissue surfaces (i.e., hydroxyl, amine, and carboxyl groups) [20, 33]. These multivalent interactions are particularly effective under hydrated conditions and enable strong, conformal adhesion to soft tissues. The shear adhesion strength to the tissue was measured to be approximately 4.4 kPa (Figure 3e). Additionally, to evaluate stability under repeated deformation, a cyclic durability test was performed by applying 10 % strain 1000 times. The adhesion strength was stably maintained during this process (Figure 3f). In an ex vivo rat brain model, robust tissue adhesion was achieved without external pressure (Figure 3g). The presence of Alg‐CA on the PPU was also effective in preserving tissue adhesion performance even under immersion in aqueous conditions (Figure 3h; Movie S1). This is attributable to the combination of the viscoelastic relaxation property of the polymer and the tissue adhesion property of the hydrogel, which enabled a more stable attachment via the rapid dissipation of the applied stress. For further confirmation, the substrate was intentionally attached in a straight line across a distinct gyrus on the bovine brain surface. Over time, the substrate gradually conformed to the curvature of the tissue; eventually, spontaneous shape morphing with close contact to the cortical surface was observed (Figure 3i; Movie S2).

### 2.3 A Brain‐Adhesive Sensor (B‐Sensor)

**FIGURE 4:**
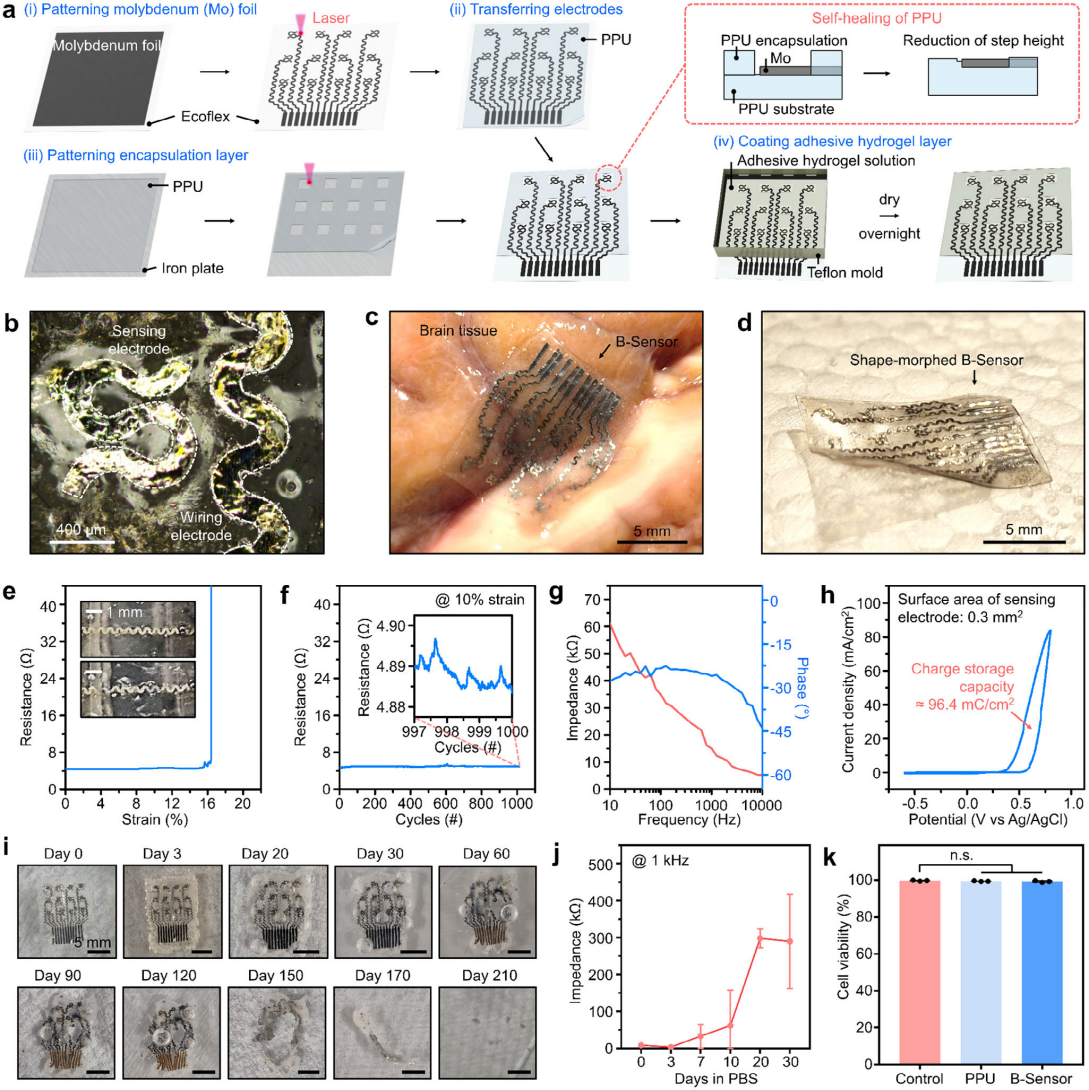
Fabrication and characterization of the B‐Sensor. (a) Fabrication steps: (i) laser patterning of molybdenum foil on Ecoflex, (ii) transfer of patterned electrodes onto PPU film, and (iii) laser‐cut PPU encapsulation layer exposing channel and interconnection regions. (b) Optical image of the completed B‐Sensor showing distinct sensing and wiring areas. (c,d) Photographs showing conformal contact of the B‐Sensor on bovine brain tissue (c) and shape‐morphed B‐Sensor (d). (e) Resistance changes during stretching. (f) Electrical stability maintained under 1000 repeated cycles of 10 % strain. (g) Electrochemical impedance spectra measured in PBS solution. (h) Cyclic voltammetry of B‐Sensor. (i) Biodegradation behavior of the B‐Sensor in 7.4 pH PBS solution at 40°C. (j) Impedance stability monitored during degradation in PBS. (k) Cell‐viability test comparing control, PPU, and B‐Sensor.

The B‐Sensor was fabricated as follows: First, a Mo foil was placed on an Ecoflex substrate, and electrode patterns were formed via laser cutting (Figure 4a‐i). The patterned Mo electrodes were then transferred to a PPU substrate (Figure 4a‐ii). In particular, the thermoplastic behavior of the PPU enables the Mo electrode array to be easily detached from the Ecoflex substrate, thereby achieving a stable transferring process. In parallel with this, the PPU film was placed on an iron plate, and a PPU encapsulation layer was patterned via laser cutting such that only the channels  were exposed (Figure 4a‐iii). Finally, the encapsulation layer obtained in Step (iii) was laminated on top of the Mo electrode array prepared in Step (ii), and the two PPU layers were stably bonded via their self‐healing property (Figure 4a, inset showing a red‐colored dashed box). Subsequently, a tissue‐adhesive hydrogel solution was poured into a Teflon mold and dried overnight to complete the final device. The Mo electrode patterns corresponding to sensing and wiring (interconnect) areas were clearly confirmed (Figure 4b). The as‐fabricated B‐Sensor exhibited 95 % stress relaxation within 60 s, a value nearly identical to that of the PPU (Figure 3b). This dynamic stress relaxation is attributed to its thin structure and serpentine geometry, which minimized mechanical constraint and allowed the bending‐induced strain energy to be effectively dissipated. As expected, the B‐Sensor achieved a high degree of conformal contact by shape‐morphing along the curvature of the bovine brain surface (Figure 4c). Even after detachment, the deformation remained consistent with cortical shape (Figure 4d).

The B‐Sensor exhibited stable deformation up to 16 % strain, ensuring mechanical compatibility with the physiological movement range of the brain (i.e., approximately 0.4 % under cardiac pulsation and up to 3 % during mild head motion) (Figure 4e) [50, 51]. Furthermore, the electrical performance was stably maintained over 1000 repeated cycles at 10 % strain (Figure 4f). To confirm nerve interfacing efficiency, electrochemical properties of the B‐Sensor were investigated in phosphate‐buffered saline (PBS) solution using a three‐electrode system (Figure 4g,h). The impedance at 1 kHz was approximately 15 kΩ, which is suitable for neural signaling [52, 53, 54]. Cyclic voltammetry measurement further showed a charge storage capacity of ∼96.4 mC cm^−2^, which is comparable to Pt, IrOx, and PEDOT:PSS (several to tens of mC cm^−2^) [55, 56]. These results suggest that the B‐Sensor can provide sufficient charge capacity and be considered for neural stimulation applications. Beyond these electrochemical characteristics, Mo‐based ECoG electrodes are known to exhibit signal acquisition performance comparable to that of conventional Pt electrodes [57].

To verify that the B‐Sensor can safely degrade in the brain without requiring a secondary surgery for removal, an accelerated degradation experiment was performed under both physiologically relevant and accelerated conditions. For physiologically relevant evaluation, the B‐Sensor was immersed in PBS at 40°C, where gradual degradation was observed over a period of approximately six months (Figure 4i). In addition, an accelerated degradation experiment was performed by immersing the B‐Sensor in a 5 m NaOH solution at 90°C. Consequently, the B‐Sensor mostly degraded after approximately 6 days (Figure S10). However, determining whether the electrical performance was maintained during the period of use is more important than confirming its complete degradation. Therefore, electrical impedance was measured under the same conditions. The B‐Sensor maintained an impedance of less than 1 MΩ at 1 kHz for 30 days (Figure 4j).

Prior to conducting in vivo experiments, the cytotoxicity of the B‐Sensor was evaluated using a cell‐viability assay. Even when the B‐Sensor was exposed to culture conditions, the cell viability remained at a level similar to that of the control group, thus confirming its excellent cytotoxicity (Figure 4k; Figure S11). Cell viability remained comparable to the control group when cells were treated with PBS containing complete degradation products of the B‐Sensor (Figure S12). These results show that the B‐Sensor simultaneously satisfied the mechanical stability, electrical reliability, and cytotoxicity requirements in biological environments. Therefore, our platform holds potential for application in implantable bioelectronic devices.

### 2.4 MRI Compatibility

**FIGURE 5:**
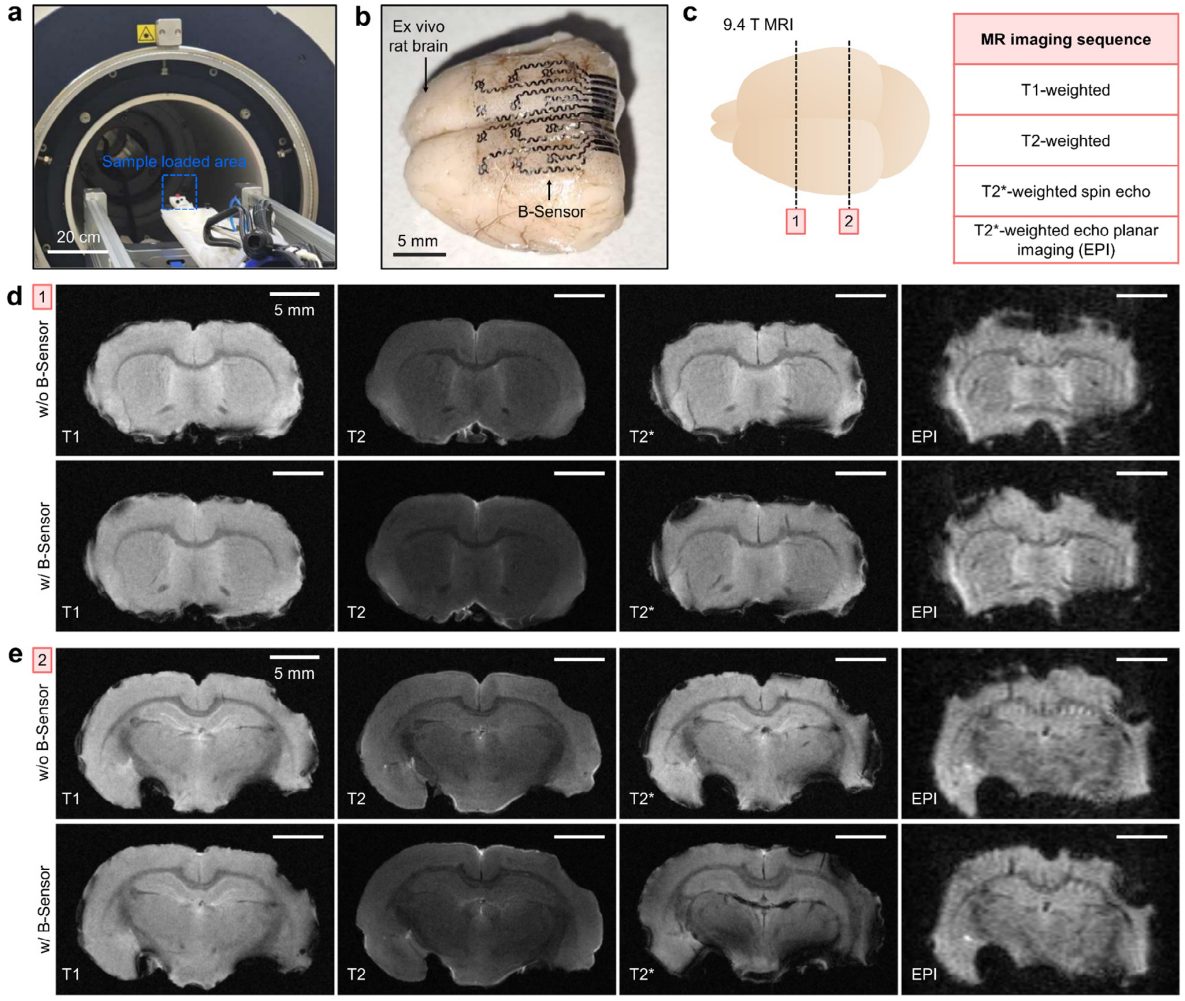
MRI compatibility of the B‐Sensor. (a,b) MRI experimental setup (a) and B‐Sensor placed on rat brain samples (b) for validation. (c) Types of MR imaging sequences. (d,e) MRI images of the rat brain with (d) and without (e) B‐Sensor showing minimal noise and artifact‐free imaging at different sites.

Generally, ferromagnetic metals such as Fe, Co, and Ni appear black during MRI measurements, thus disturbing brain‐structure imaging due to their magnetic susceptibility [58]. Therefore, non‐ferromagnetic metals such as Mo, Au, Pt, and Ti can be adopted [59, 60]. Despite imaging compatibility, when thick and bulky metallic materials are used, tissue damage may occur owing to heat generated by induced currents. To overcome the challenges, applying the thin Mo electrode with the wavy and open‐mesh design in neural implants is desirable for compatibility with MRI operation. From this standpoint, MRI measurements were performed by placing electrodes on ex vivo rat brain tissue inside the 9.4T MRI (Figure 5a,b). In clinical settings, T1‐weighted imaging is mainly used to visualize anatomical structures clearly, while T2‐weighted imaging is useful for detecting pathological changes such as edema or lesions [61].

T2^*^‐weighted imaging is widely applied to assess metal‐induced artifacts due to its pronounced sensitivity to magnetic field inhomogeneity [62]. Notably, T2^*^‐weighted echo planar imaging (EPI) enables rapid acquisition, making it suitable for functional MRI applications [63]. To simulate various clinical scenarios, imaging was performed using these multiple modes (Figure 5c). The B‐Sensor yielded almost no noise and provided clear MRI images without any blacked‐out regions (Figure 5d,e; Figures S13 and S14). In addition, no apparent MRI‐induced heating effects were observed. The brain surface showed no noticeable tissue damage or discoloration after MRI scanning, indicating the absence of significant thermal injury during imaging (Figure S15).

### 2.5 Site‐Specific Somatosensory Evoked Potential Responses Using the Multichannel B‐Sensor

**FIGURE 6:**
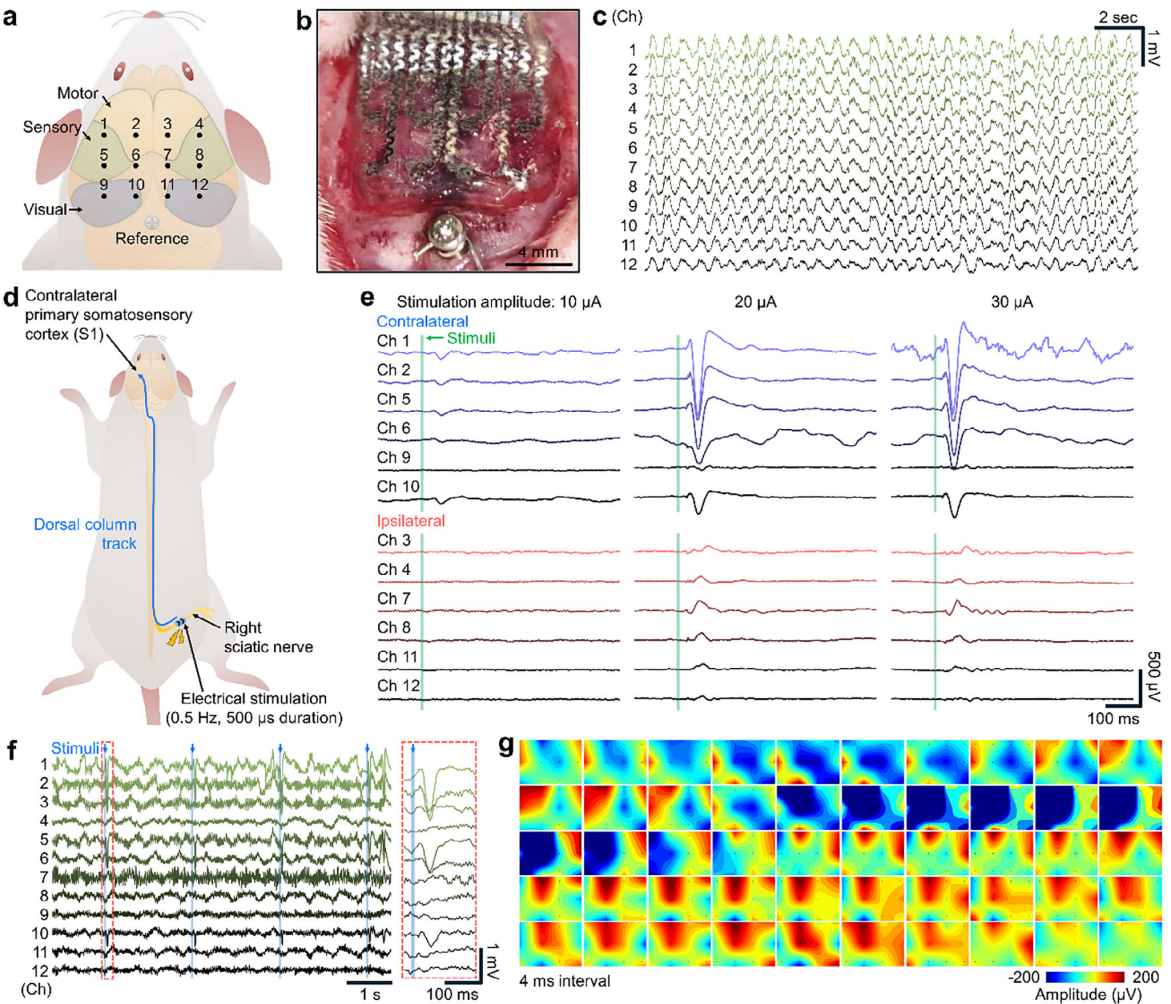
Somatosensory evoked potential (SEP) recording using the B‐Sensor. (a) Schematic showing the 12‐channel electrode layout on the rat cortex. (b) Photograph of the B‐Sensor implanted on the cortical surface. (c) Baseline neural signals recorded under ketamine anesthesia. (d) Experimental setup for SEP measurement with electrical stimulation to the right sciatic nerve. (e) SEP waveforms recorded under weak stimulation (left), mild stimulation (middle), and strong stimulation (right). (f) Raw 12‐channel SEP recordings showing distinct responses across channels. (g) Topographical map of SEP responses indicating localized activation in the contralateral sensorimotor cortex.

The B‐Sensor was implanted on the cerebral cortex of a rat to perform neural signal‐recording experiments. The electrodes were arranged in a 12‐channel array covering the visual, motor, and sensory cortices (Figure 6a,b). Under ketamine anesthesia, baseline signals were first measured (Figure 6c), which confirmed that the electrodes in the B‐Sensor could stably record neural activity.

Next, somatosensory evoked potential (SEP) measurements were performed to evaluate channel‐specific responses. Electrical stimulation (10‐30 µA, 500 µs pulse duration, 0.5 Hz) was applied to the right sciatic nerve at an intensity set slightly above the motor threshold—defined as the minimal current required to induce visible muscle twitching—or slightly below this level to avoid excessive activation. All twelve‐channel signals were simultaneously recorded using the B‐Sensor (Figure 6d). Because the applied stimulus primarily elicited sensory afferent signals, the evoked responses were expected to propagate through the dorsal column pathway and be recorded in the contralateral somatosensory cortex [31, 64]. Relatively small SEP amplitudes (i.e., 100 µV in channel 1) were observed under weak stimulation (10 µA), whereas strong stimulation (30 µA) yielded markedly larger amplitudes (i.e., 1196 µV in channel 1) (Figure 6e), reflecting the graded nature of neural recruitment with increasing stimulus intensity. In line with this intensity‐dependent behavior, channels 1, 2, 5, and 6 in the somatosensory region exhibited prominent signals that scaled with stimulus strength. As shown in the raw 12‐channel recordings (Figure 6f), distinct temporal and spatial activity patterns were observed across all channels. Subsequent spatial mapping of these signals (Figure 6g) revealed localized activation in the contralateral sensorimotor cortex, consistent with the expected topographical organization of somatosensory processing.

### 2.6 Spatiotemporal ECoG Mapping of Seizure Dynamics With the B‐Sensor

**FIGURE 7:**
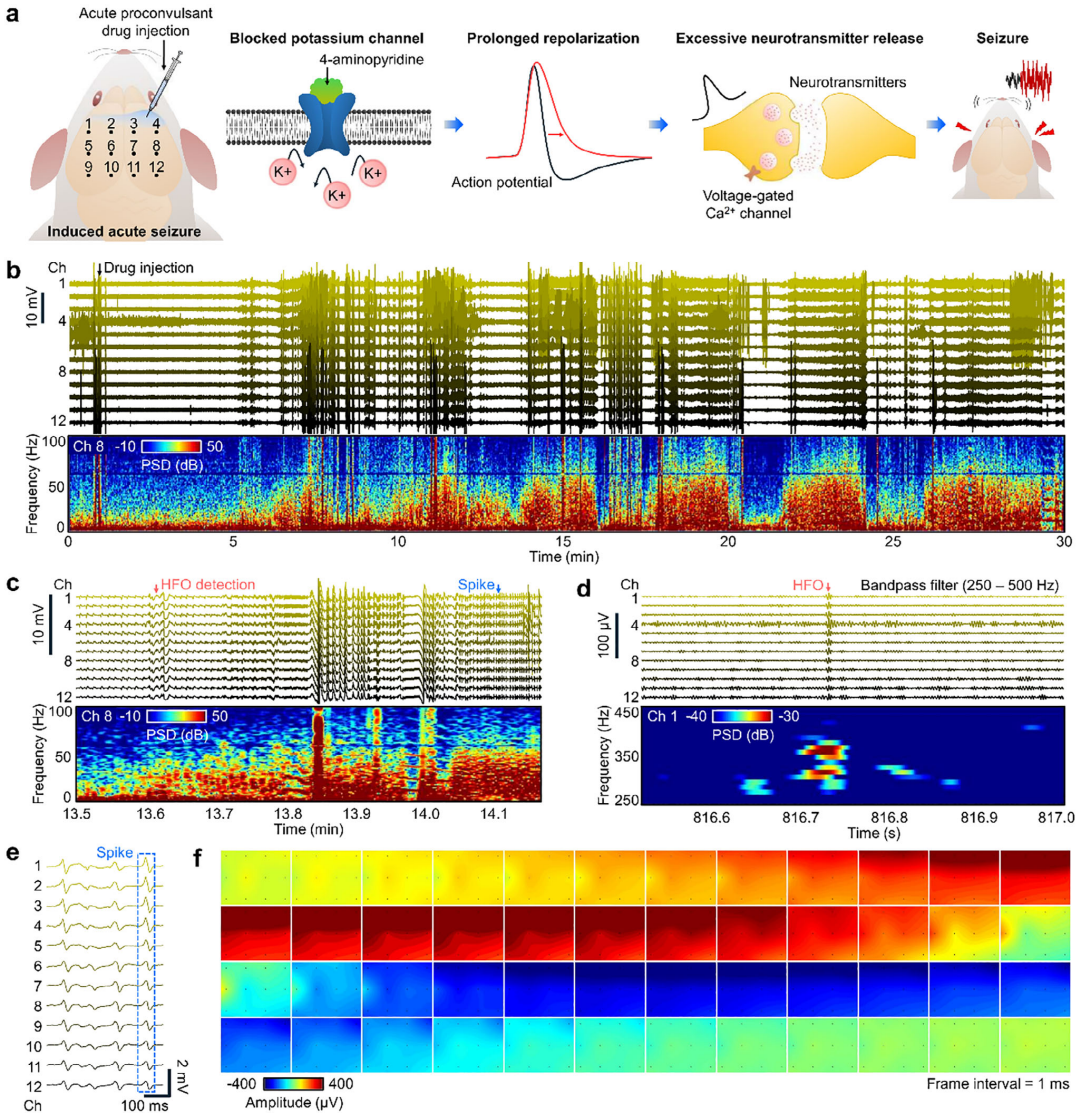
Spatiotemporal mapping of seizure Dynamics with the B‐Sensor. (a) Schematic showing the 12‐channel electrode layout on the rat cortex and 4‐aminopyridine (4‐AP) administration for the drug‐induced epilepsy model. (b) Full‐scale 12‐channel ECoG recording and spectrogram of epileptiform activity following 4‐AP injection. (c) Magnified 12‐channel ECoG recording and spectrogram. (d) Detection of high‐frequency oscillations (HFOs). (e) Representative spikes showing distinct epileptiform activity across cortical sites. (f) Spatiotemporal mapping of a single spike.

To confirm the feasibility of precise diagnosis, a drug‐induced epilepsy model was established by administering 4‐aminopyridine (4‐AP) to the upper right cortex (Figure 7a). 4‐AP is a well‐known proconvulsant drug that blocks voltage‐gated potassium (K^+^) channels, thereby inhibiting the normal repolarization of action potentials [65]. As a result, the duration of the action potential is prolonged, and the depolarized state is sustained, while the open time of presynaptic voltage‐gated calcium (Ca^2^^+^) channels is extended, leading to increased calcium influx. Elevated calcium levels enhance the probability of synaptic vesicle release, causing excessive neurotransmitter discharge [66, 67]. These cumulative changes drive the cortical network into a state of hyperexcitability and ultimately give rise to seizure‐like discharges. Importantly, as long as 4‐AP remains pharmacologically active, this hyperexcitable state persists, resulting in repeated ictal and interictal events that recapitulate the characteristic patterns of clinical epilepsy models.

Real‐time ECoG recordings obtained over 30 min with the B‐Sensor clearly revealed repetitive ictal and interictal discharges (Figure 7b). Spectrogram analysis of the signals in channel 8 showed that seizures were not confined to a narrow frequency range but instead manifested as broadband power increases extending up to 80 Hz, indicating the recruitment of widespread cortical networks during seizures. Magnified traces of the same interval (Figure 7c) provided detailed temporal information of the epileptiform discharges, which could be classified into tonic and clonic phases [68]. The tonic phase corresponded to a broadband seizure pattern, characterized by sustained high‐amplitude discharges across multiple frequency bands, reflecting prolonged depolarization and widespread cortical hyperexcitability. In contrast, the clonic phase was defined by rhythmic repetitive discharges in which a fundamental frequency and its harmonics were clearly observed, appearing as high‐amplitude field transients recurring periodically with progressively increasing inter‐burst intervals. This tonic–clonic transition closely mimics the hallmark features of generalized tonic–clonic seizures observed clinically and demonstrates that 4‐AP can induce heterogeneous seizure dynamics within the same experimental paradigm.

In addition, high‐frequency oscillations (HFOs) were detected during the recordings (Figure 7d). The HFO has recently been recognized as a clinically relevant indicator for the early identification of epileptogenic zones and as a potential predictor of seizure onset [69, 70]. The detection of HFOs in the 4‐AP induced seizure model indicates that the B‐Sensor is highly sensitive not only to low‐frequency seizure discharges but also to high‐frequency components not accessible by conventional EEG, with translational value for early diagnosis. The HFO spectrograms were well isolated and clearly captured, confirming reliable measurement. Furthermore, 12‐channel ECoG data obtained from the multichannel B‐Sensor revealed distinct seizure patterns across different cortical sites (Figure 7e) [71]. Spatiotemporal topographical mapping of a single spike showed that seizures originated at the site of drug application and progressively propagated over time into surrounding cortical regions (Figure 7f; Movie S3). This pattern of focal onset followed by cortical spread recapitulates typical propagation pathways observed in seizures [70]. Collectively, these results demonstrate that the B‐Sensor can successfully record both normal physiological and drug‐induced pathological activities with high spatiotemporal resolution, capturing seizure onset, frequency characteristics, phase transitions, HFOs, and spatiotemporal propagation with high fidelity.

## 3 Conclusion

In this study, our B‐Sensor with nerve adhesion, biodegradability, and shape adaptability was newly developed. To impart unique capabilities to the B‐Sensor, we first synthesized the self‐healing and biodegradable PPU with low *T*_g_ and rapid viscoelastic relaxation properties. The Mo electrodes, fabricated using the laser‐driven patterning technique, were then integrated into the PPU substrate. After applying the PPU encapsulation layer, the nerve‐adhesive Alg‐CA hydrogel was coated onto the Mo electrode array fully supported on the PPU layer, thus allowing reliable electrical operation under repeated mechanical loading as well as conformal contact even with fine cortical curvatures. Biodegradation tests also demonstrated that the B‐Sensor disappeared naturally under specific conditions after a certain period, thus confirming the advantage of avoiding removal surgery for short‐term monitoring. Cell‐viability assays further confirmed its excellent cytotoxicity, with results comparable to those of the control group. Additionally, the use of molybdenum‐based open‐mesh electrodes significantly reduced heating and imaging artifacts under MRI conditions, and artifact‐free images were successfully obtained in ex vivo rat brain experiments.

Functional validation showed that the B‐Sensor reliably recorded both normal and pathological neural activities. In SEP experiments, channel‐specific ERP responses were distinguished based on muscle stimulation, and the localized activation of the sensorimotor cortex was clearly mapped. In the 4‐AP‐induced epilepsy model, the epileptiform discharge synchronization was observed in the low‐frequency range, and spatial propagation patterns were precisely captured. These findings show that the B‐Sensor functions as a high‐resolution platform capable of revealing both physiological and pathological neural dynamics, beyond simple signal recording. Expanding the resolution and channel density of the device and integrating wireless and real‐time data transmission are expected to further enhance its potential for long‐term monitoring and clinical translation.

In clinical settings, subdural ECoG electrode grids with approximately 10 mm inter‐electrode spacing are commonly used. While this configuration is sufficient for routine clinical evaluation, there is a growing trend toward high‐density electrode arrays with 2–4 mm spacing to enable more precise neural recordings and finer delineation of localized pathological features. Within this context, the B‐Sensor adopts a denser electrode configuration with an effective spacing of 2.5 mm, enabling higher‐resolution cortical mapping while maintaining mechanical compliance. Future developments will focus on expanding channel count and array dimensions, as well as integrating wireless and real‐time data transmission, to further assess the feasibility of translating the proposed platform to human electrocorticography applications

## 4 Experimental Section/Methods

### 4.1 Materials

Bis‐MPA (>98 %, Tokyo Chemical Industry), 1‐bromopropane (99 %, Sigma–Aldrich), ethyl chloroformate (97 %, Sigma), benzyl bromide (99 %, Thermo Fisher Scientific), triethyamine (99 %, Daejung Chemicals), potassium hydroxide (KOH, 93 %, Daejung Chemicals), benzoic acid (99 %, Alfa Aesar), 4,4′‐methylenebis(phenyl isocyanate) (MDI, 98 %, Sigma–Aldrich), and DBTDL (95 %, Sigma–Aldrich) were utilized. 1,8‐Diazabicyclo[5.4.0]undec‐7‐ene (DBU, 98 %, Sigma–Aldrich) was dried over CaH_2_ and distilled before use. All solvents, including acetonitrile (MeCN), tetrahydrofuran (THF), dimethylformamide (DMF), and dichloromethane (DCM), were purchased from Daejung Chemicals.

### 4.2 Characterization

The chemical structure of monomers and polymers (PC‐diols, PPU) was observed by ^1^H and ^13^C NMR (Bruker, Avance III HD 400 MHz) using chloroform‐d (CDCl_3_). Chemical shifts were referenced to solvent resonance signals. Fourier transform infrared (FT‐IR) spectra were recorded over the wavenumber range 4000–800 cm^−1^ via an FT‐IR spectrometer (Thermo Fisher Scientific Inc., Nicolet iS50). Size exclusion chromatography (SEC) was used to record number average molecular weight (*M*_n_) and polydispersity index (Ð, *M*_w_/*M*_n_) of the polymer by a Waters SEC system equipped with a refractive index detector (RI‐2414) using DMF (0.05 m LiBr) as the mobile phase at 50°C with a flow rate of 0.5 mL min^−1^. The samples were separated by four columns (Styragel HR2, Styragel HR 4, Styrage HR 4E, Styragel Guard Column). A polymer solution was prepared at a concentration of ca. 3 mg/mL, and 50 µL was injected. The molecular weight was calibrated based on a polymethylmethacrylate (PMMA) standard. Thermal gravimetric analysis (TGA) was performed using a TA Instrument TGA Q50‐1200 under N_2_ atmosphere at a temperature ramp rate of 10°C/min. To measure glass transition temperatures of synthesized polymers, differential scanning calorimetry (DSC) was performed with a TA Instrument DSC Q20‐1426 using a heating and cooling rate of 10°C/min from −60°C to 100°C under N_2_ atmosphere.

### 4.3 Degradable Polymer Synthesis

#### 4.3.1 Synthesis of propyl 3‐hydroxy‐2‐(hydroxymethyl)‐2methylpropanoate (Propyl MPA)

In a one‐neck RBF (250 mL), Bis‐MPA (15 g, 0.11183 mmol, 1 equivalent) and trimethylamine (17 mL, 0.12301 mol, 1.1 equiv.) were dissolved in acetonitrile (100 mL) and stored at 80°C for 1 h. Subsequently, allyl bromide solution (13 mL, 0.14538 mmol, 1.30 equivalent) and acetonitrile (12 mL) were added slowly to the reaction flask and continuously stirred at 70°C overnight. The reaction solution was cooled to RT, and salt was generated. The white salt was removed via vacuum filtration. The filtrate was concentrated and further purified by column chromatography on silica gel using a mixture of ethyl acetate and hexane (8:2, v/v) as the eluent. The product obtained was a viscous yellow liquid. (Yield: 12.1 g, 61.4 %). ^1^H NMR (400 MHz, CDCl_3_, ppm) was performed under δ: 4.12 (t, *J* = 6.6 Hz, 2H), 3.90 (d, *J* = 11.2 Hz, 2H), 3.70 (d, *J* = 11.2 Hz, 2H), 2.78 (s, 2H), 1.69 (hept, *J* = 6.8 Hz, 2H), 1.06 (s, 3H), and 0.95 (t, *J* = 7.4 Hz, 3H). ^13^C NMR (101 MHz, CDCl_3_, ppm) was performed under δ: 176.07, 67.67, 66.63, 49.29, 21.98, 17.24, and 10.40.

#### 4.3.2 Synthesis of MPC

In a one‐neck RBF (500 mL), propyl‐MPA (12.10 g, 0.06867 mol, 1 equivalent) and ethyl chloroformate (20 mL, 0.206000 mol, 3 equivalents) were dissolved in THF (140 mL). The mixture was stirred at 0°C for 30 min. Triethylamine (29 mL, 0.20600 mol, 3 equivalents) was added dropwise into the flask over 30 min. The ice bath was removed, and the reaction was stirred at RT overnight. After the salt was removed, the filtrate was concentrated and purified on silica gel via column chromatography. The eluent used for separation was the mixture of ethyl acetate and hexane at a ratio of 2:8. The resulting product was a slightly yellow, viscous liquid. (Yield: 7.88 g, 56.8 %). ^1^H NMR (400 MHz, CDCl_3_, ppm) was performed under δ: 4.63 (d, *J* = 10.9 Hz, 2H), 4.17 (d, *J* = 10.9 Hz, 2H), 4.10 (t, *J* = 6.6 Hz, 2H), 1.63 (hepta, *J* = 7.3 Hz, 2H), 1.26 (s, 3H), and 0.89 (t, *J* = 7.5 Hz, 3H). ^13^C NMR (101 MHz, CDCl_3_, ppm) was performed under δ: 171.21, 147.60, 73.02, 67.68, 40.17, 21.78, 17.43, and 10.17.

#### 4.3.3 Synthesis of Bn‐MPA

In a two‐neck RBF (250 mL), the solution of Bis‐MPA (20 g, 0.14911 mol, 1 equivalent) and KOH (9.54 g, 0.16998 mol, 1.14 equivalent) dissolved in DMF (115 mL) was stored at 100°C for 1 h. Benzyl bromide (21.33 mL, 0.17938 mol, 1.2 equivalent) was added slowly to the reaction flask and stirred continuously at 100°C for 15 h. The reaction solution was concentrated, dissolved in DCM, and extracted twice with deionized (DI)‐water. The organic layer was then dried over MgSO_4_, filtered, and concentrated. The crude extract was mixed with 10 mL of DCM, poured into hexane (90 mL), and stored in a freezer for 2 h. White crystals were obtained by filtration and drying under vacuum. (Yield: 23.9 g, 71.5 %).^1^H NMR (400 MHz, CDCl_3_, ppm) was performed under δ: 7.42–7.29 (m, 5H), 5.21 (s, 2H), 3.94 (d, *J* = 11.2 Hz, 2H), 3.74 (d, *J* = 11.3 Hz, 2H), 2.88 (s, 2H), and 1.08 (s, 3H). ^13^C NMR (101 MHz, CDCl_3_, ppm) was performed under δ: 175.89, 135.80, 128.8, 128.48, 128.02, 68.65, 66.86, and 17.60.

#### 4.3.4 Synthesis of MBC

In a two‐neck RBF (250 mL), Bn‐MPA (20 g, 0.08918 mol, 1 equivalent) and ethyl chloroformate (25.5 mL, 0.26755 mol, 3 equivalent) were dissolved in THF (200 mL). The mixture was stirred at 0°C for 30 min. Triethylamine (37.3 mL, 0.26755 mol, 3 equivalents) was added dropwise into the flask over 30 min. The ice bath was removed, and the reaction was continued at RT overnight. After removing the salt, the filtrate was concentrated, dissolved in THF (50 mL), and poured into the diethyl ether (450 mL). After being stored in a freezer for 2 h, the white crystals were retrieved and dried in a vacuum. (Yield: 13.62 g, 53.1 %). ^1^H NMR (400 MHz, CDCl_3_, ppm) was performed under δ: 7.42–7.30 (m, 5H), 5.22 (s, 2H), 4.71 (d, *J* = 10.9 Hz, 2H), 4.22 (d, *J* = 10.9 Hz, 2H), and 1.34 (s, 3H). ^13^C NMR (101 MHz, CDCl_3_, ppm) was performed under δ: 170.05, 147.53, 134.90, 128.91, 128.89, 128.36, 73.08, 68.06, 40.38, and 17.73.

#### 4.3.5 Synthesis of Polycarbonate‐Diol Via Organocatalytic Ring‐Opening Polymerization of Cyclic Carbonate Monomers (PC‐diol)

In a two‐neck RBF (50 mL), Bn‐MPA (0.35 g, 1.56 mmol, 1 equivalent), MPC (1.58 g, 7.8 mmol, 5 equivalents), and MBC (1.56 g, 6.24 mmol, 4 equivalents) were placed and dried overnight under vacuum. After evacuating the vacuum, dried DCM (8 mL, 2 m) was added and subsequently bubbled with N_2_ for 30 min. DBU (0.5 mL, 3.12 mmol, 2 equivalents) was added to the monomer/initiator solution. After 2 h, polymerization was quenched by adding a benzoic acid (0.57 g, 4.6821 mmol, 3 equivalent) solution in DCM (5 mL, 1 m). PC‐diol was purified via silica gel column chromatography using a mixture of ethyl acetate and hexane (4:6 to 6:4, v/v) as the eluent. PC‐diol was obtained as a colorless, viscous liquid. (Yield: 3.07 g, 88 %). ^1^H NMR (400 MHz, CDCl_3_, ppm) was performed under δ: 7.33 (br, –OCH_2_C_6_ ***H***_5_), 5.15 (s, –OC ***H***_2_C_6_H_5_), 4.62 (br, –OC ***H***_2_CHCH_2_), 4.28 (br, –OC(O)OC ***H***_2_–), 4.08 (br, –OC ***H***_2_CH_2_CH_3_), 3.70 (m, –C ***H***_2_OH), 1.63 (br, —‐OCH_2_C ***H***_2_CH_3_), 1.24 (s, –C(C ***H***_3_)(CH_2_O–)_2_), and 0.91 (br, —‐OCH_2_CH_2_C ***H***_3_). ^13^C NMR (101 MHz, CDCl_3_, ppm) was performed under δ: 172.06, 154.49, 135.53, 131.64, 128.72, 128.48, 128.12, 118.67, 68.75, 67.21, 67.08, 46.72, 21.98, 17.54, and 10.39.

#### 4.3.6 Synthesis of PPU via Polycondensation Reactions

In a two‐neck RBF (25 mL), PC‐diol (3.07 g, 1.7483 mmol, 1 equivalent) and MDI (0.44 g, 1.7483 mmol, 1 equivalent) were dissolved in DCM (6 mL) and bubbled with N_2_ for 30 min. The mixture was continuously stirred at RT for 24 h after adding DBTDL (0.2 mL, 0.2972 mmol, 0.17 equivalent). After the mixture was precipitated in methanol, decanted, and dried overnight in a vacuum at RT, a polymer appearing as a sticky white solid was obtained (yield: 2.99 g, 82.3 %). ^1^H NMR (400 MHz, CDCl_3_, ppm) was performed under δ: 7.31‒7.08 (br, –NHC_6_ ***H***_5_), 7.06 (br, –OCH_2_C_6_ ***H***_5_), 5.14 (s, –OC ***H***_2_C_6_H_5_), 4.28 (br, –O(C═O)OC ***H***_2_–), 4.08 (t, –(C═O)OC ***H***_2_–), 3.87 (s, –C_6_H_5_C ***H***_2_C_6_H_5_–), 1.59 (m, –CH_2_C ***H***_2_CH_3_), 1.24 (s, –C(C ***H***_3_)(CH_2_O–)_2_), and 0.91 (t, –CH_2_CH_2_C ***H***_3_). ^13^C NMR (101 MHz, CDCl_3_, ppm) was performed under δ: 172.06, 154.53, 135.53, 129.55, 128.73, 128.49, 128.28, 128.13, 68.76, 67.21, 67.08, 46.72, 21.98, 17.61, and 10.40.

### 4.4 Preparation of PPU Films and Mechanical Testing

To evaluate mechanical properties, PPU was processed into films. Specifically, 7 g of PPU was completely dissolved in 30 mL of DCM and then cast into a Teflon mold measuring 20 cm in diameter, followed by natural solvent evaporation. The resulting film had a thickness of approximately 400 µm. The film was cut into specimens measuring 1 cm × 3 cm. At both ends of each specimen (1 cm × 1 cm), paper and double‐sided 3 m tape were attached to form handles. Tensile testing was performed using a universal testing machine (UTM; Instron 34SC‐1, Instron) at a rate of 20 mm min^−1^, and the stress was recorded as a function of strain. The Young's modulus was calculated from the slope of the stress–strain curve in the 1 %–2 % strain range. For the stress‐relaxation test, the strain of 30 % was applied at a rate of 20 mm min^−1^, and stress changes were monitored for 10 min. The self‐healing property was assessed by cutting the sample, allowing it to heal at room temperature for 8 h, and subsequently measuring its performance.

### 4.5 Tissue Adhesive Strength

To evaluate tissue adhesion, the surface of the PPU film was coated with an Alg‐CA hydrogel. Alg‐CA hydrogel was synthesized following the method described in a previous study [33]. Prior to coating, the PPU film was treated with oxygen plasma. An Alg‐CA solution (in DI water) was then applied and dried. The coated samples were cut into 1 cm × 1 cm pieces and attached to an OHP film using double‐sided 3 m tape. Rat dorsal skin was excised into 1 cm × 1 cm pieces with the inner surface exposed and then fixed onto the OHP film using an instant adhesive. Subsequently, the Alg‐CA‐coated PPU and skin tissues were placed in contact, and the OHP film handles were mounted on the UTM. Tensile testing was performed at a rate of 50 mm/min, and the maximum load was divided by the contact area (1 cm^2^) to calculate the adhesion strength.

### 4.6 B‐Sensor Fabrication

PPU films were prepared by dissolving 1 g of PPU in 30 mL of DCM and casting the solution into a Teflon mold measuring 20 cm in diameter, followed by solvent evaporation. Separately, an Ecoflex (Ecoflex 00–30, Smooth‐on) substrate (4 cm × 4 cm, cured on a glass mold) was used as a support for a 1 µm‐thick molybdenum foil (GoodFellow). The molybdenum electrode was patterned via laser cutting through an optical fiber laser marker (HY‐F10, Hyosung Laser) and subsequently transferred onto a PPU film.

For encapsulation, a PPU film was placed on an iron plate and laser cut to expose only the electrode channel and interconnected areas. The electrode‐transferred PPU film and encapsulated PPU layer were then laminated and bonded together by exploiting the self‐healing property of PPU. Finally, the Alg‐CA hydrogel was poured into a square Teflon mold, dried, and used to complete the tissue‐adhesive layer of the device.

### 4.7 Ex Vivo Brain Tissue Conformability Study

The bovine cerebellum was dissected into slices and maintained at 40°C under continuous saline irrigation to preserve tissue hydration. Alg‐CA–coated PPU films and B‐Sensor were placed on the tissue surface, and their adhesion was further examined after immersion in PBS. Conformability to the curved tissue surface was qualitatively monitored for up to 1 h.

### 4.8 Electrical Characterization

The electrical properties of the electrodes were evaluated before encapsulation and after electrode transfer onto the PPU substrate. The samples were mounted on an automatic stretcher, connected through liquid‐metal contacts, and wired to a digital multimeter (Keithley 2450 source meter, Tektronix) using copper leads. A voltage of 1 V was applied, and the current was measured to calculate the resistance based on Ohm's law. The fracture behavior was assessed by monitoring the resistance during stretching at a rate of 3 mm min^−1^. Fatigue resistance was further tested by applying 10 % strain repeatedly for 1000 cycles.

### 4.9 Electrochemical Characterization

Impedance and cyclic voltammetry measurements were performed using the completed B‐Sensor immersed in PBS solution. The electrode was connected as the working electrode, with an Ag/AgCl electrode and a Pt mesh serving as the reference and counter electrodes, respectively. Impedance was measured using a potentiostat (ZIVE SP1, ZIVE LAB) with a three‐electrode setup in the frequency range of 1–10000 Hz. Cyclic voltametric measurement was conducted in the potential window of −0.65–0.8 V at a scan rate of 100 mV s^−1^. Current density was obtained by normalizing the measured current to the exposed electrode area.

### 4.10 Device Degradability

The degradability of the B‐sensor was evaluated under physiologically relevant conditions by immersing the device in PBS at 40°C. Device degradation was monitored over time by visual inspection until complete disappearance. For comparison, an accelerated degradation test was performed by immersing the B‐sensor in 5 m NaOH at 90°C, and degradation behavior was similarly monitored.

### 4.11 Cell Viability

To evaluate the cytocompatibility of the PPU and B‐Sensor, mouse fibroblast cells (L929) were precultured in growth media (Dulbecco's modified Eagle medium (low glucose, Gibco)) supplemented with 10 v/v % fetal bovine serum (Gibco) and 1 v/v % penicillin–streptomycin (Gibco). The releasates from the samples were collected in Dulbecco's modified Eagle medium (5 mL per 10 × 13 mm^2^ patch sample) for 24 h at 37°C. The cells (10 000 cells per well) were seeded in a 24‐well plate. After 42 h, the growth medium was replaced with the 5 fold diluted release (1 mL). In addition, for the evaluation of the cytocompatibility of complete degradation products, the B‐Sensor was fully degraded in PBS under physiologically relevant conditions. The resulting PBS solution containing degradation products was diluted tenfold with Dulbecco's modified Eagle medium and then applied to the cells. For the 72 h cultures, cell viability was determined using the live/dead assay (Invitrogen). Fluorescent images were obtained using a Leica fluorescence microscope (DMi8, Leica), and the green dots for live cells and red dots for dead cells were counted using ImageJ software.

### 4.12 MR Imaging

MR imaging was performed using a rodent MR scanner (Bruker 9.4 T) to obtain T1‐, T2‐, T2^*^‐, and EPI‐weighted images. Ex vivo rat brains were prepared with the B‐Sensor placed on the cortical surface and without the sensor as a control.

### 4.13 In Vivo Experiments

All animal experiments were approved by the Institutional Animal Care and Use Committee (IACUC no. SKKUIACUC2025‐02‐45‐2 and SKKUIACUC2025‐09‐50‐1). Male Sprague–Dawley rats at 8 weeks of age were used for the experiments.

To record brain signals, the animals were anesthetized with ketamine, and their scalp and hind limb hair were removed. The head was fixed in a stereotaxic frame, and the scalp was incised to expose the skull. A craniotomy was performed using a hand drill to expose the cerebral cortex, and a reference screw was inserted into the cerebellum. The B‐Sensor was placed on the cortical surface. Neural signals were acquired at a sampling rate of 30 kHz using data acquisition (Cerebus, Blackrock Microsystems).

SEP measurements were performed by inserting a stimulation cuff electrode around the right sciatic nerve. Electrical pulses were delivered at 2 s intervals by a stimulator (Model 2100 Isolated Pulse Stimulator, A‐M Systems), and TTL signals were recorded simultaneously to synchronize the stimulation timing. To induce epileptiform activity, 25 mM 4‐AP was prepared in saline and injected locally into the cerebral cortex using a 0.5 mL insulin syringe (0.5 cc, BD Ultra‐Fine). Epileptic signals were recorded for 1 h, during which seizures were confirmed by behavioral manifestations, such as whisker twitching and limb tremors.

For MR imaging, the animals were anesthetized with urethane (1.88 mg kg^−1^), and their scalp and hind limb hair were removed. The head was fixed in a stereotaxic frame, and the scalp was incised to expose the skull. Craniotomy was performed using a hand drill to expose the cerebral cortex. The B‐Sensor was placed on the cortical surface, and MR imaging was subsequently performed under the conditions described above. After completion of the MRI scan, the brain tissue was harvested and processed for histological analyses to evaluate potential MRI‐induced tissue effects.

### 4.14 Histological Analysis

Harvested brain tissues were fixed in 4 % paraformaldehyde. The samples were then processed, embedded, and sectioned according to standard histological procedures. Hematoxylin and eosin (H&E) staining was performed to evaluate general tissue morphology and structural integrity. Terminal deoxynucleotidyl transferase dUTP nick end labeling (TUNEL) staining was conducted to assess potential apoptosis or cell death induced by MRI exposure. The stained sections were examined using optical microscopy for qualitative histological evaluation.

### 4.15 Signal Processing

All signals were sampled at 30 kHz. Raw signals were preprocessed using a second‐order Butterworth bandpass filter (1–550 Hz) and a 60 Hz notch filter. For SEP analysis, signals from 100 repeated stimulations were aligned to TTL triggers, and the 1 s intervals before and after stimulation were averaged to calculate the ERP. For PSD analysis, the frequency components were extracted using a 1 s window with 19/20 overlap or 0.1 s window with 99/100 overlap. The activation topography was generated by visualizing spatial activity with spatiotemporal delays based on the onset of firing in specific channels.

## Conflicts of Interest

The authors declare no conflicts of interest.

## Supporting information




**Supporting File**: advs74446‐sup‐0001‐SuppMat.docx.

**Supporting Movie**: advs74446‐sup‐0002‐MovieS1.docx.

**Supporting Movie**: advs74446‐sup‐0003‐MovieS2.docx.

**Supporting Movie**: advs74446‐sup‐0004‐MovieS3.docx.

## Data Availability

The data that support the findings of this study are available from the corresponding author upon reasonable request.
