# Supplementary material for: Brain‐Adhesive Bioelectronics With Shape‐Morphable and Biodegradable Properties for Stable Brain Signal Monitoring
Source: Adv Sci (Weinh). 2026 Mar 3;13(24):e18255. doi: 10.1002/advs.202518255 (PMC13116226; doi:10.1002/advs.202518255)
Supplement: Supplementary file 1 — Supporting File: advs74446‐sup‐0001‐SuppMat.docx. [file ADVS-13-e18255-s001.docx]

Supporting Information

Brain-Adhesive Bioelectronics with Shape-Morphable and Biodegradable Properties for Stable Brain Signal Monitoring

Heewon Choi, Soeun Kim, Sumin Kim, Jaehyun Park, Soojung An, Sungjun Yoon, Mikyung Shin, Sangho Cho* and Donghee Son*


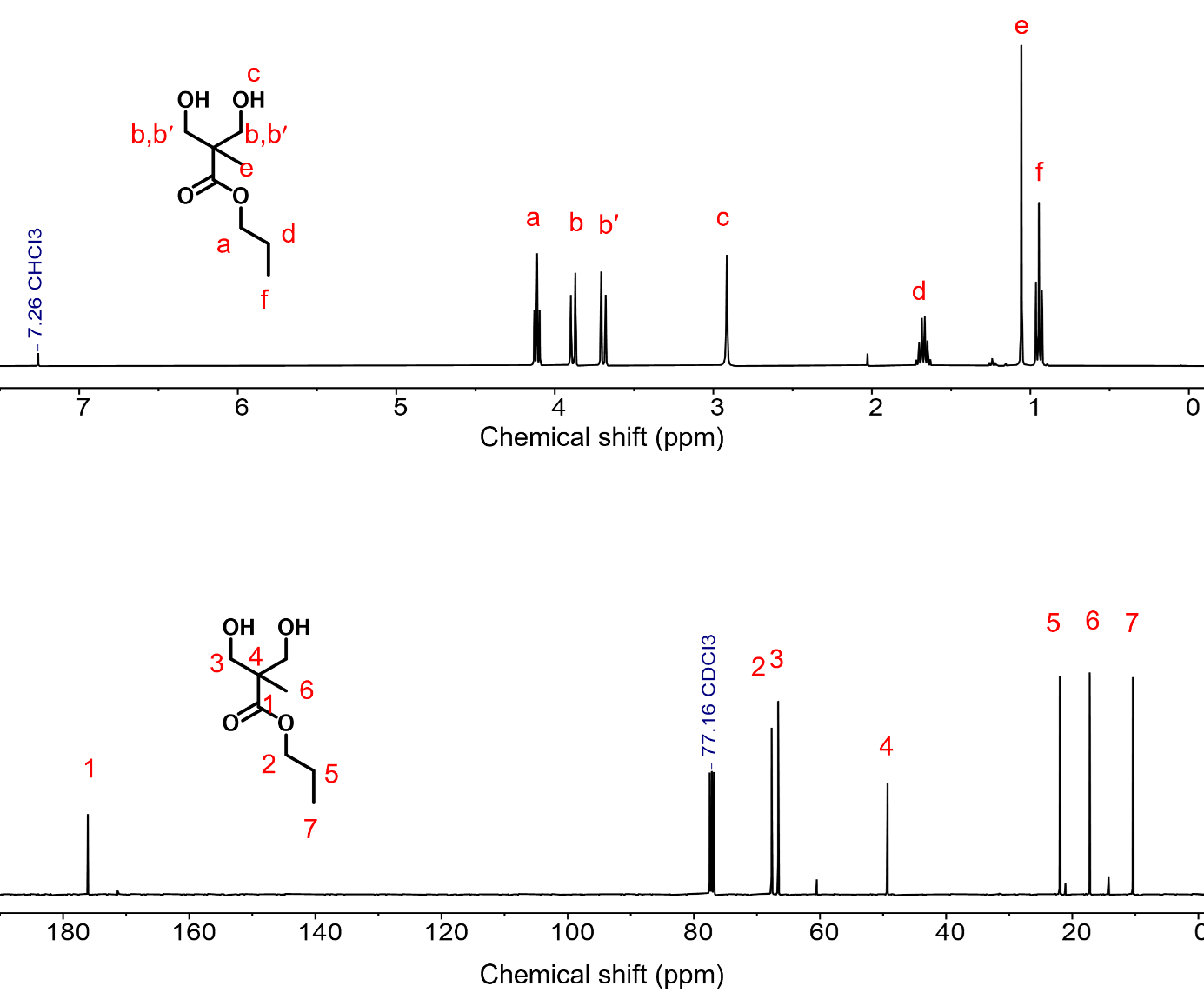
**Figure S1.** ^1^H and ^13^C NMR spectra (CDCl_3_) of Propyl (methylol)propionic acid (MPA).


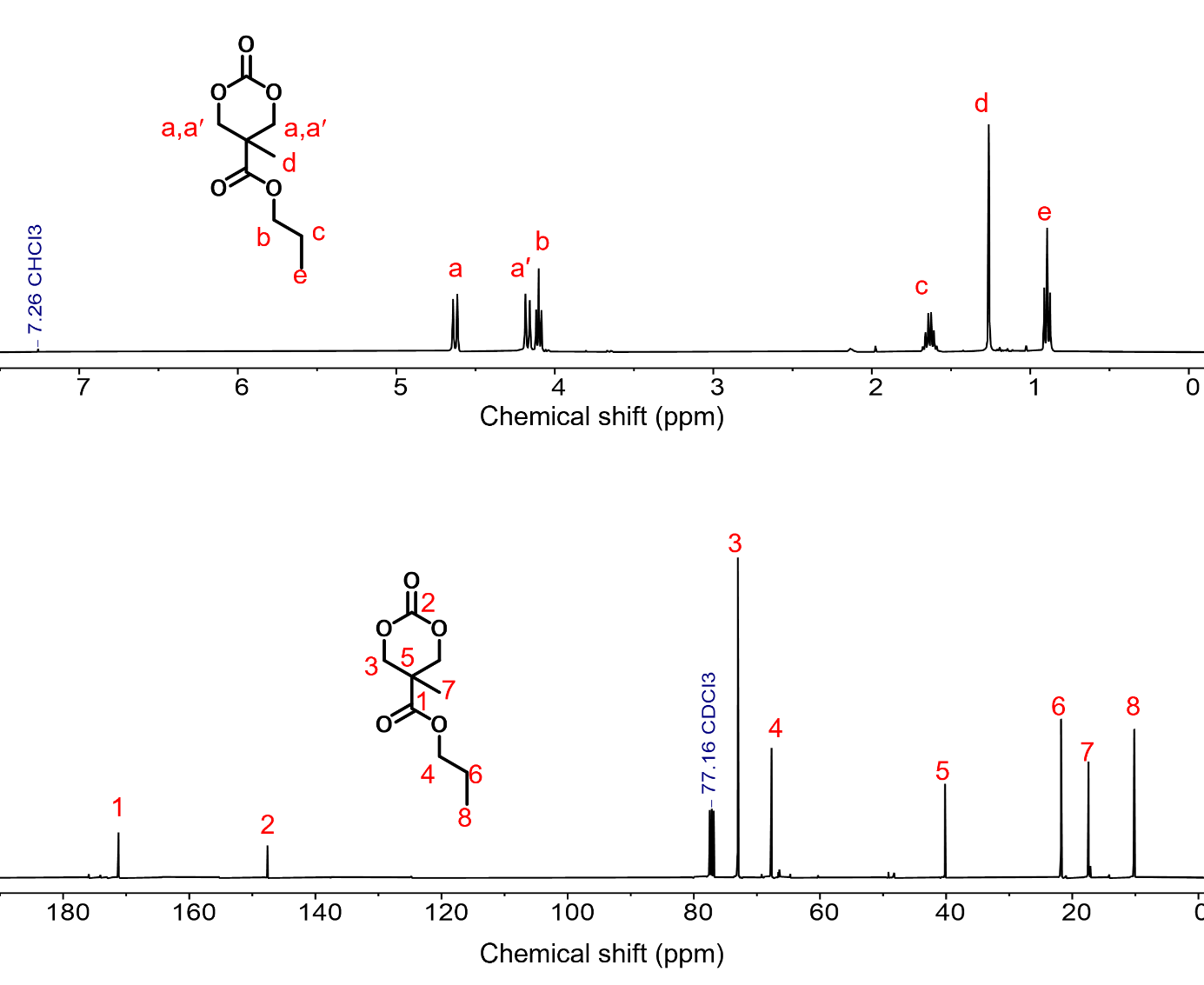


**Figure S2.** ^1^H and ^13^C NMR spectra (CDCl_3_) of propyl-functionalized cyclic carbonate (MPC).


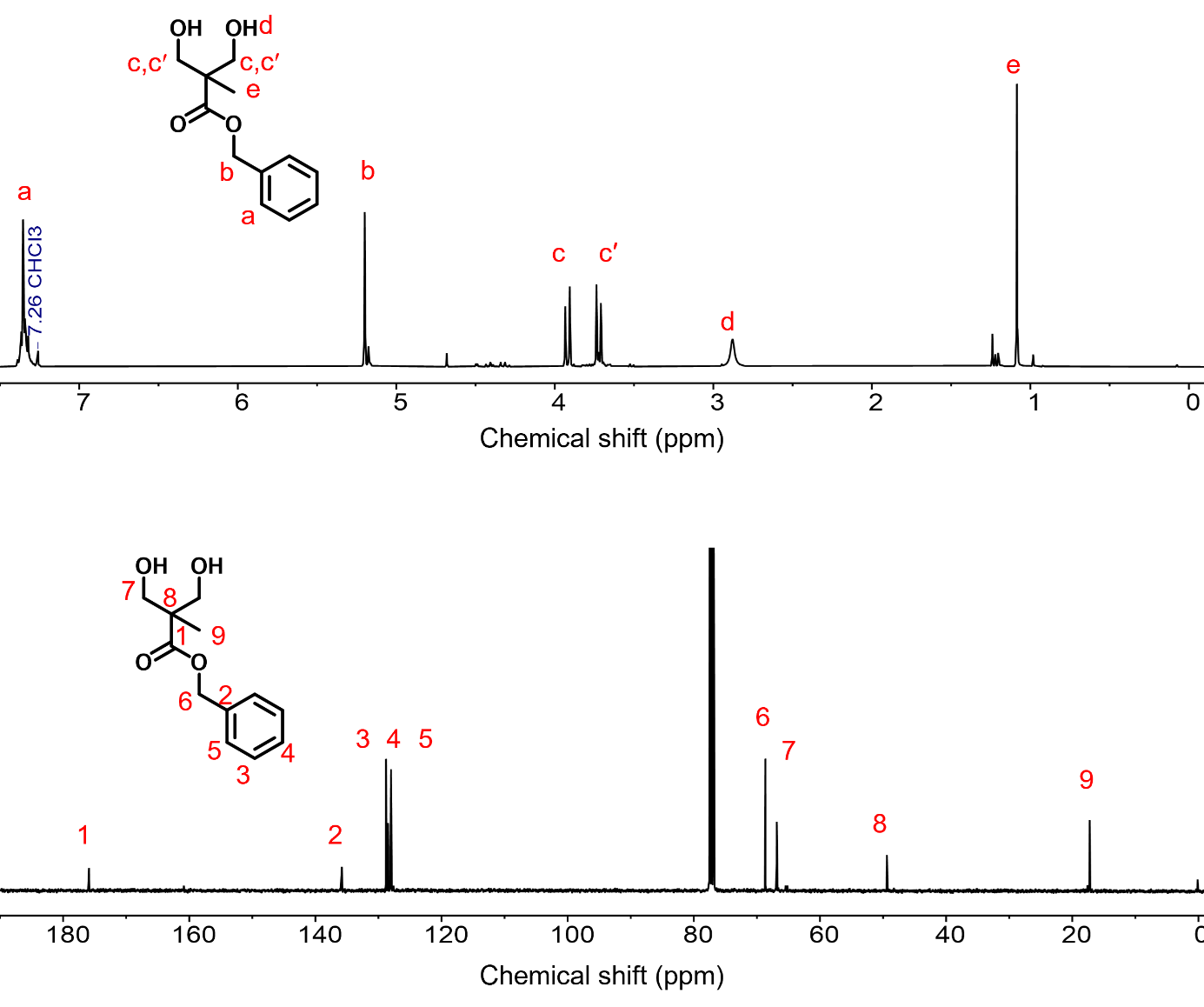


**Figure S3**. ^1^H and ^13^C NMR spectra (CDCl_3_) of benzyl 3-hydroxy-2-(hydroxymethyl)-2-methylpropanoate (Bn-MPA).


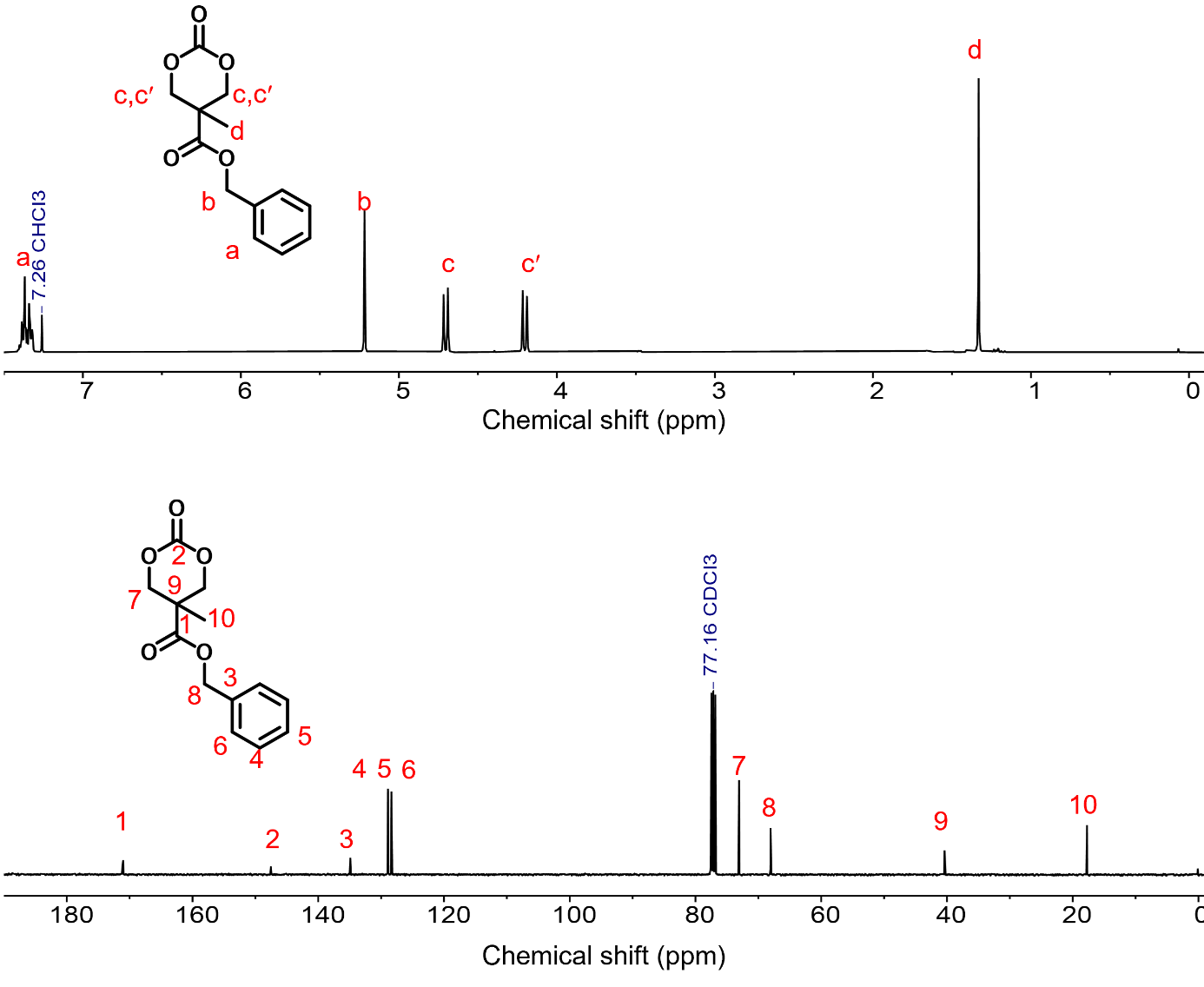


**Figure S4.** ^1^H and ^13^C NMR spectra (CDCl_3_) of benzyl-functionalized cyclic carbonate (MBC).


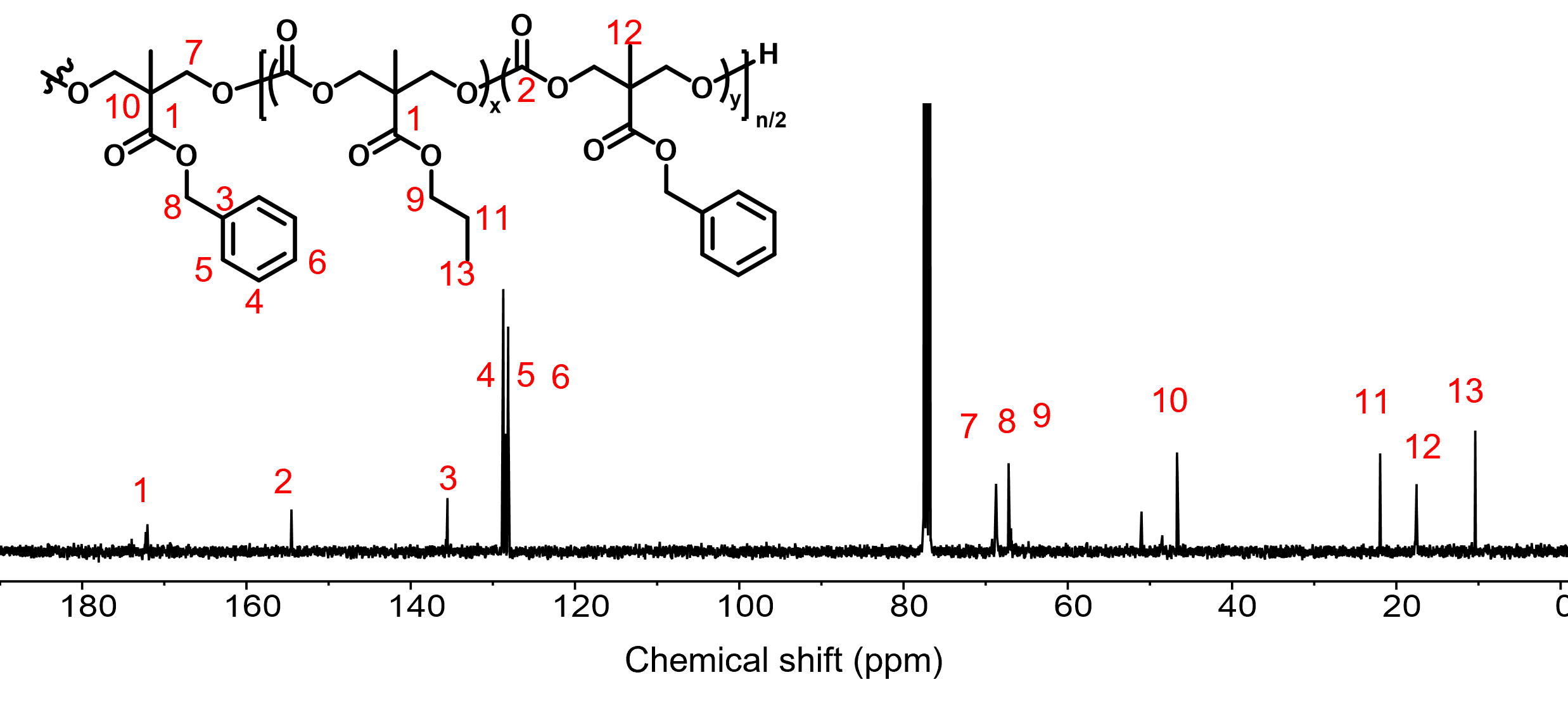


**Figure S5.** ^13^C NMR spectrum (101 MHz, CDCl_3_) of polycarbonate diol (PC-diol).


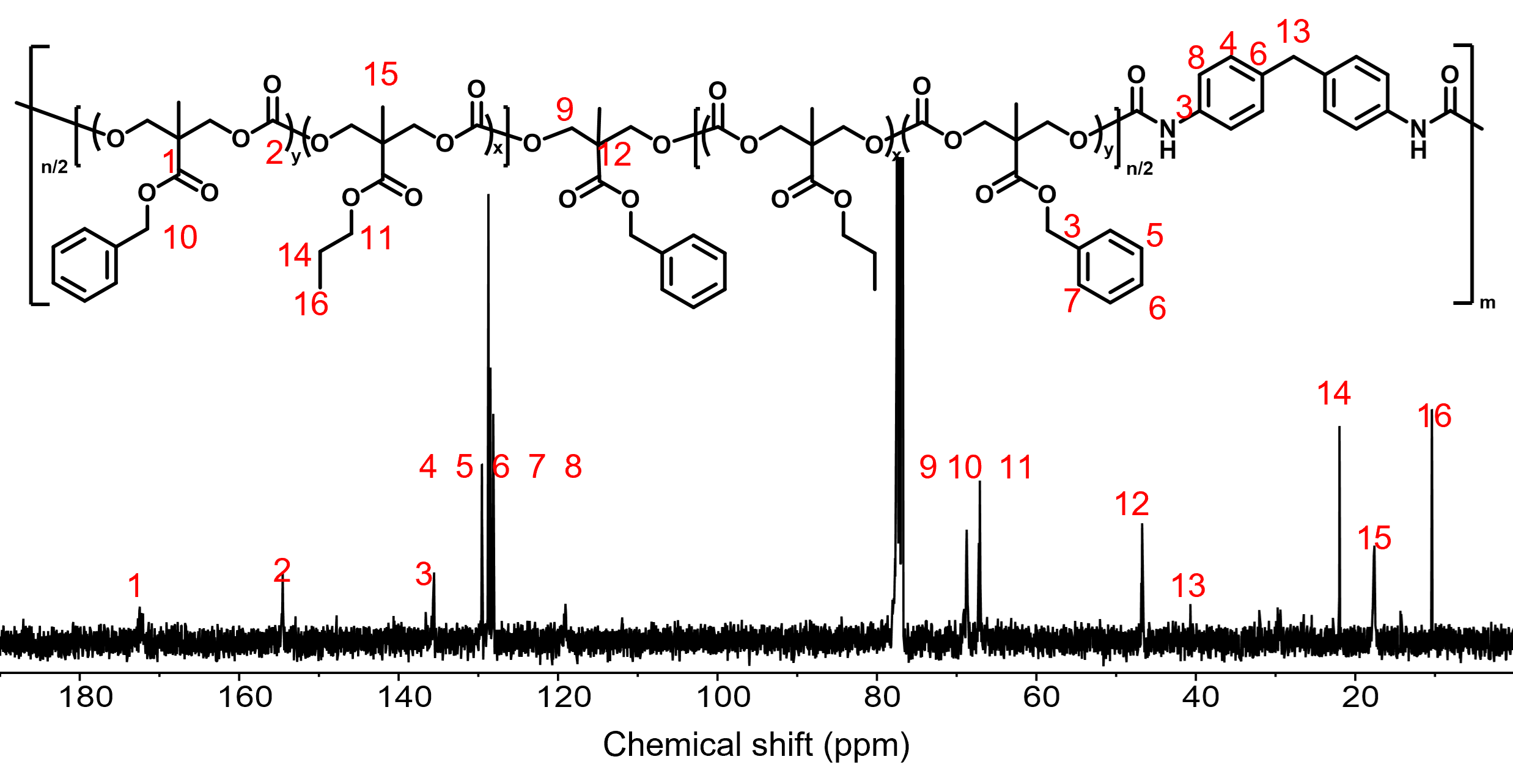


**Figure S6.** ^13^C NMR spectrum (101 MHz, CDCl_3_) of polycarbonate-based polyurethane (PPU).


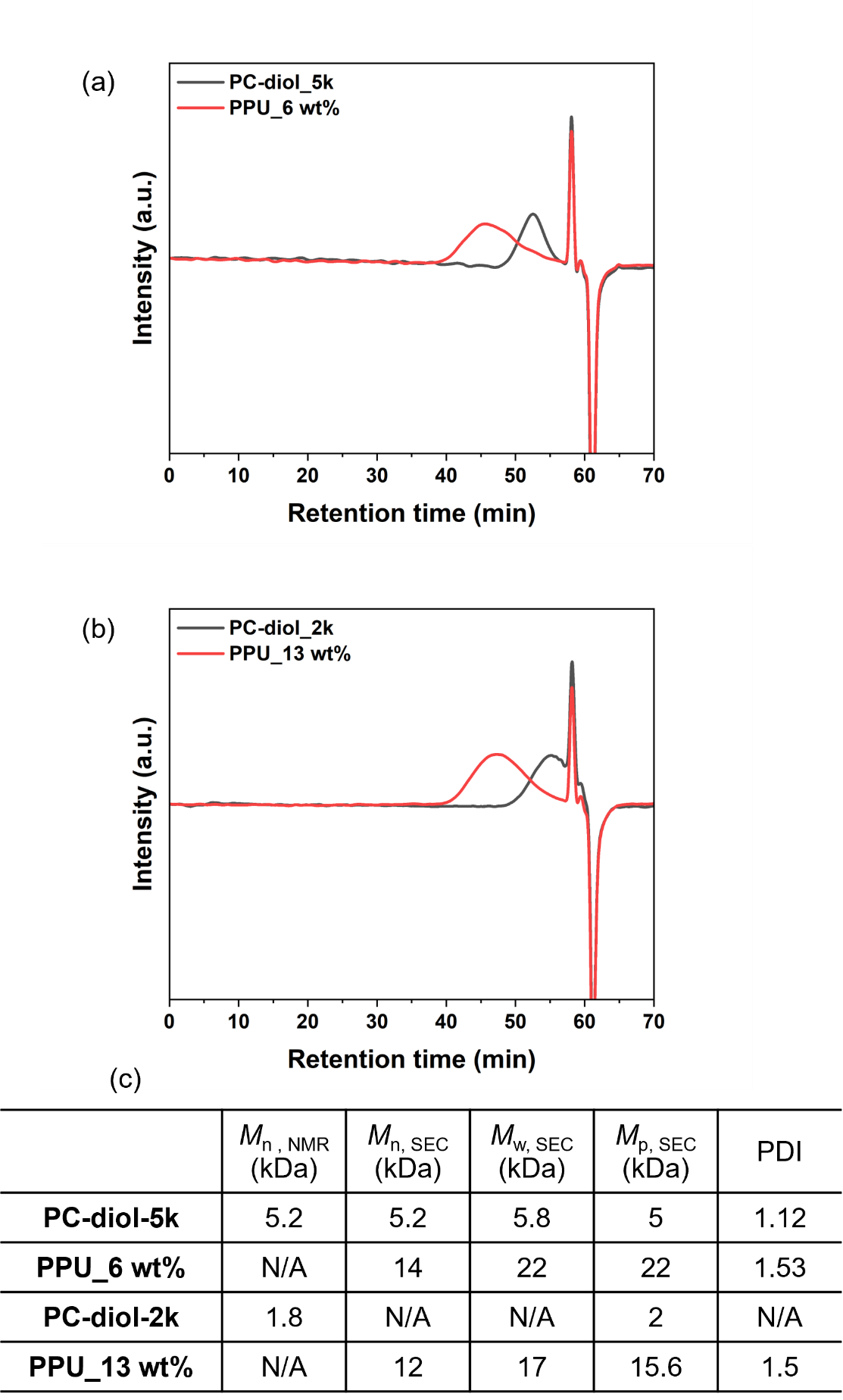


**Figure S7.** a-c) Size exclusion chromatography (SEC) traces of PC-diol and PPU (a, b), and their tabulated dispersity and mola mass values (c).


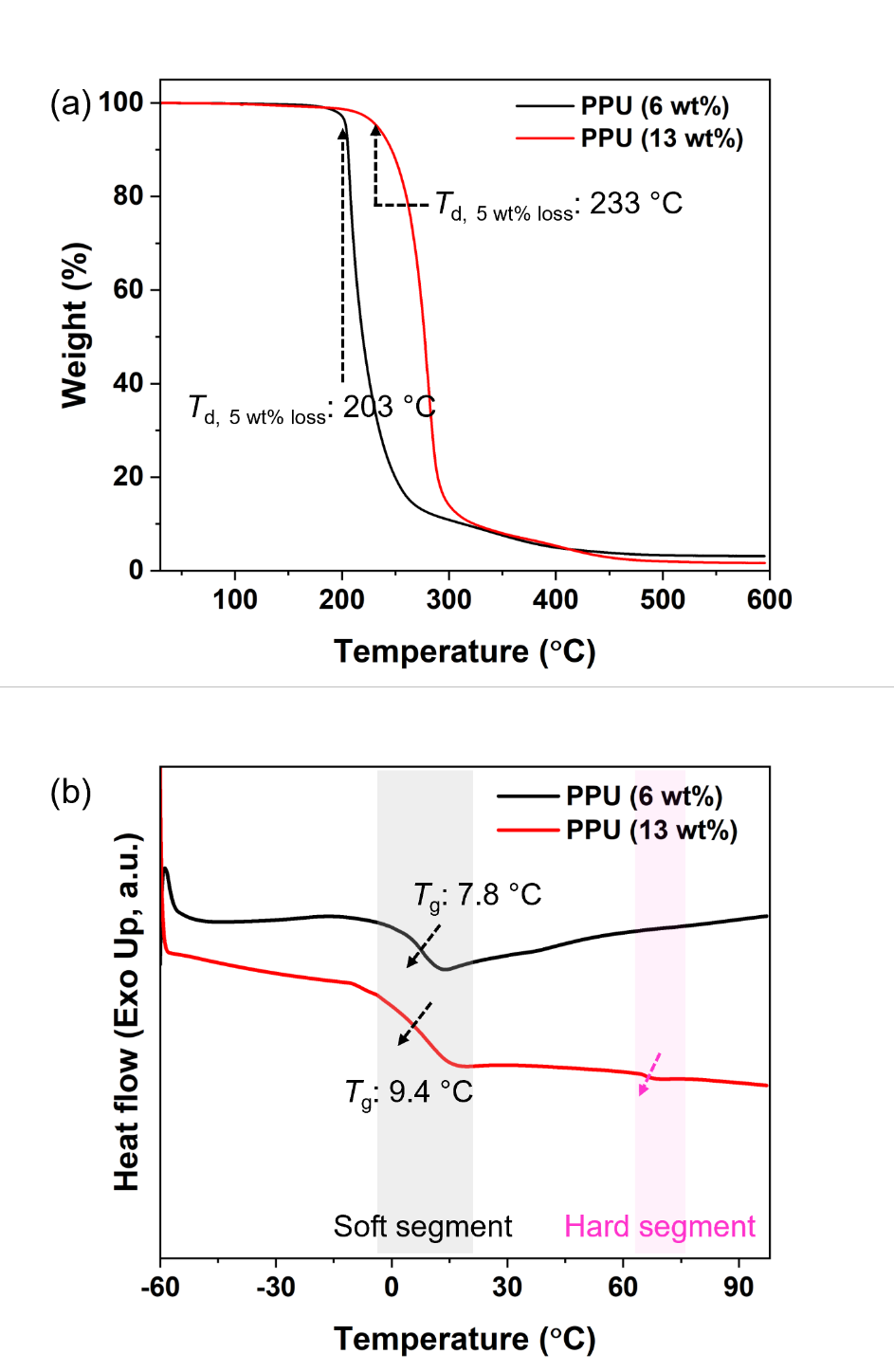


**Figure S8.** a) Thermal gravimetric analysis (TGA) curve indicating that *T*_d_ of PPU-6wt%, and 13 wt% are about 203 °C, and 233 °C, respectively. b) Differential scanning calorimetry (DSC) curve showing *T*_g_ of PPU-6wt% and 13 wt%.

**
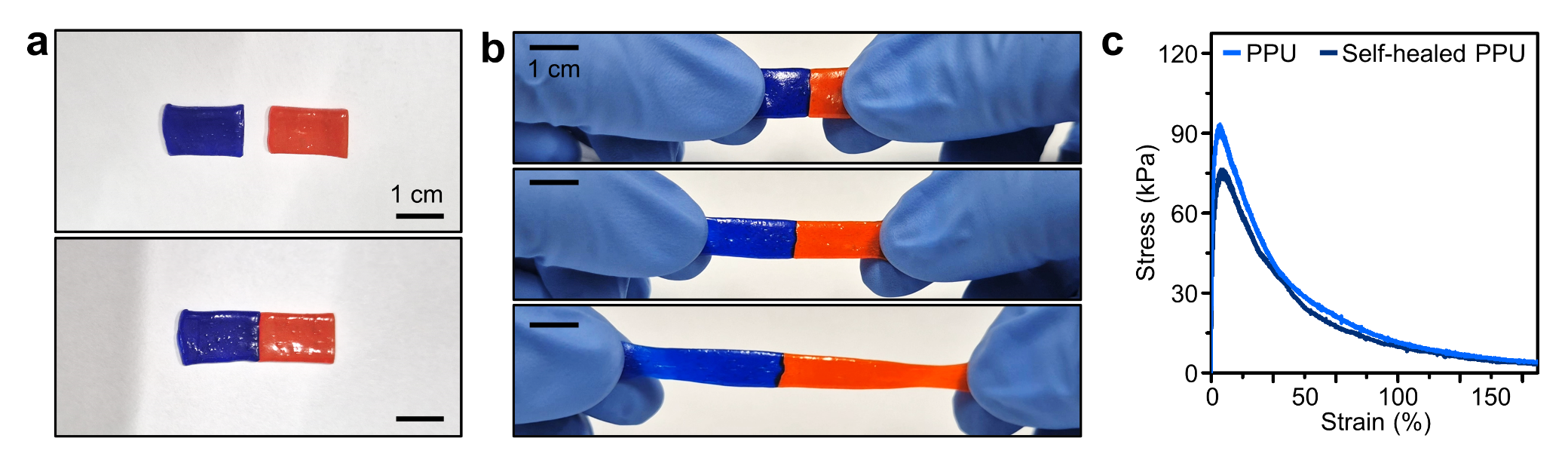
**

**Figure S9.** a) Photographs of two separate elastomer pieces and rejoined sample. b) Stretching of the self-healed sample after 8 hours at room temperature. c) Strain-stress curve of PPU before cut and after self-healing.


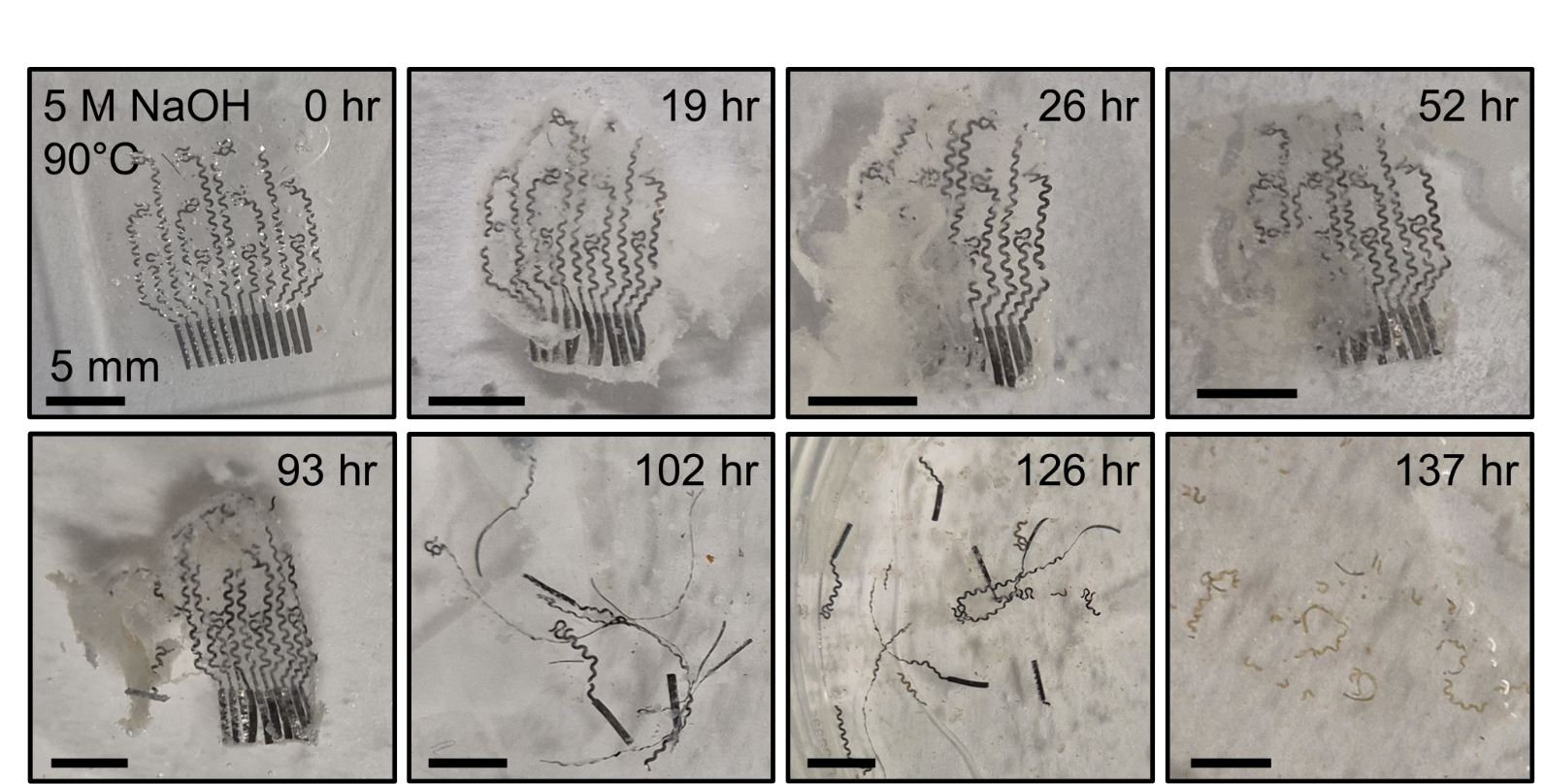


**Figure S10.** Biodegradation behavior of the B-Sensor in 5M NaOH solution at 90 °C.


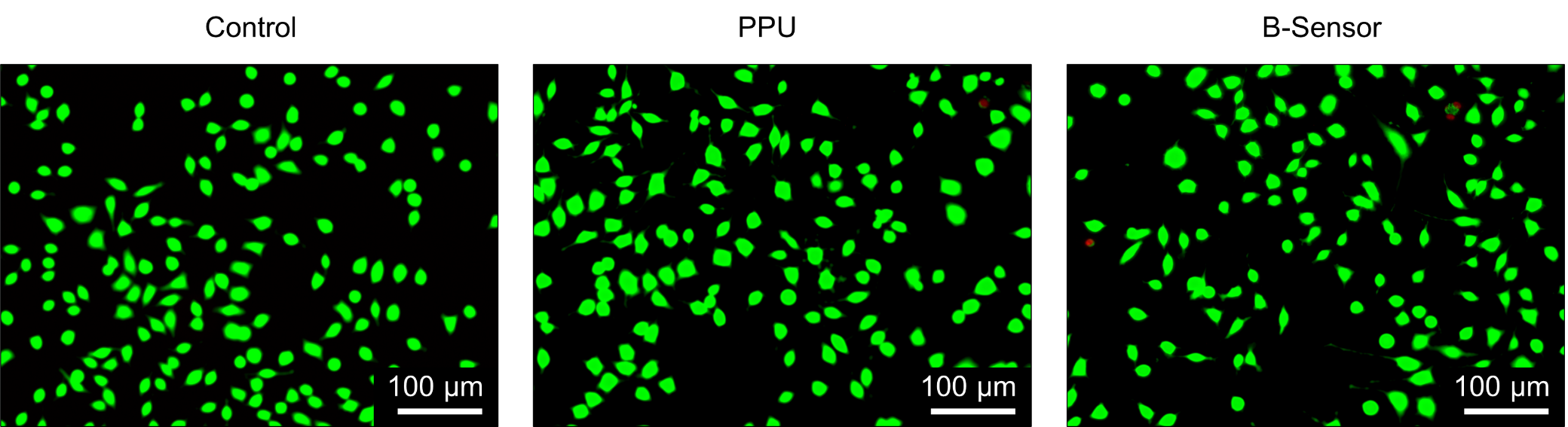


**Figure S11.** Cell viability images of control, PPU, and B-Sensor.


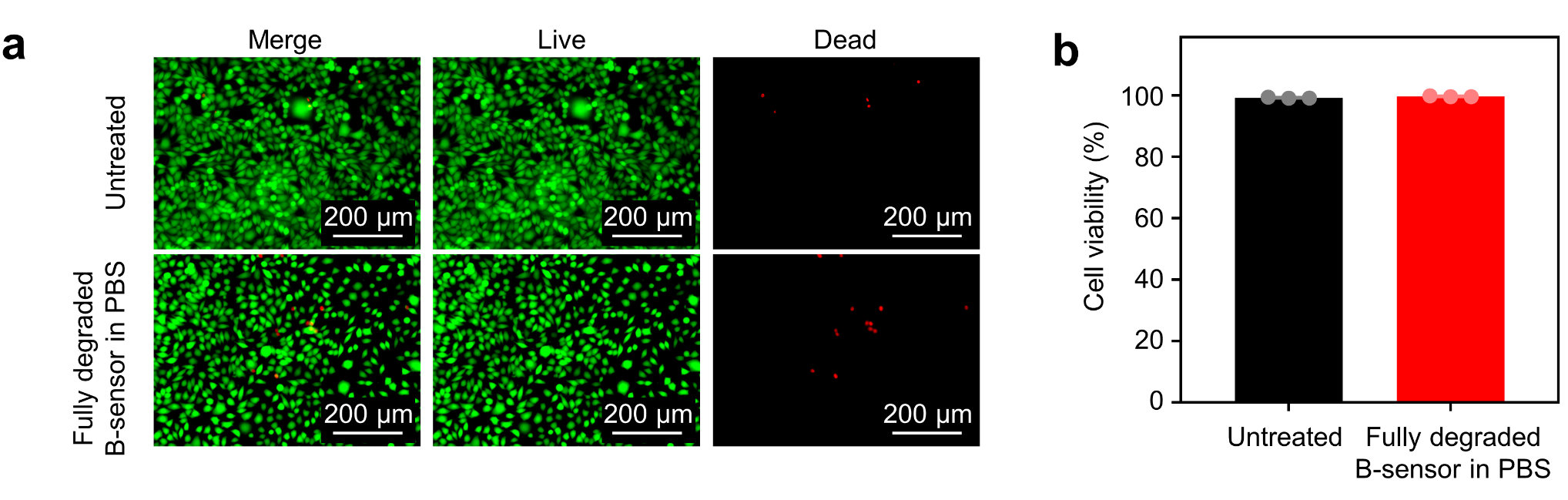


**Figure S12.** **Cytocompatibility evaluation of B-sensor degradation products.** a) Representative fluorescence images of L929 fibroblast cells in the untreated control group and after exposure to PBS containing complete degradation products of the B-sensor. B) Quantitative analysis of cell viability.


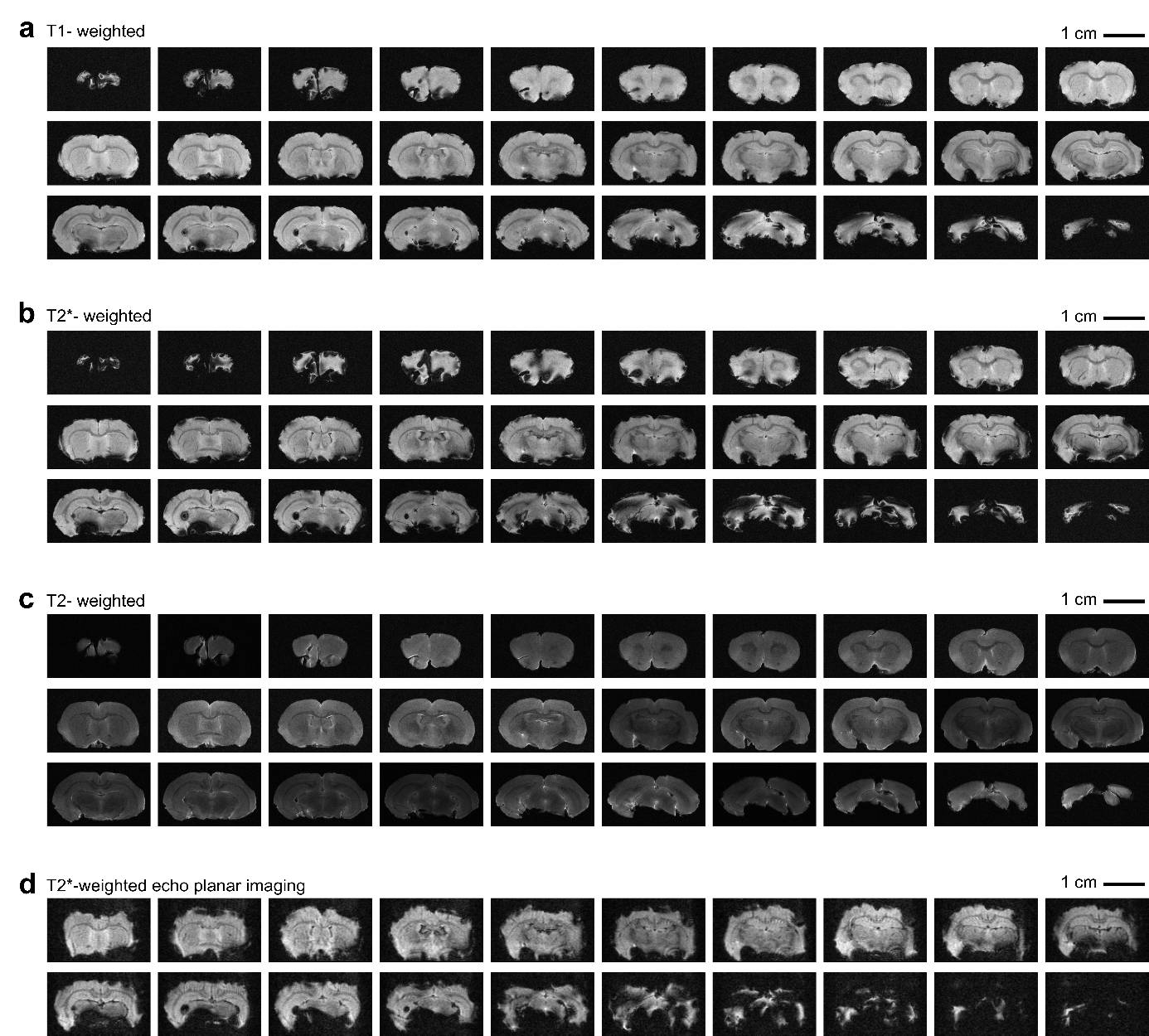


**Figure S13.** MRI images of ex vivo rat brain without B-Sensor.


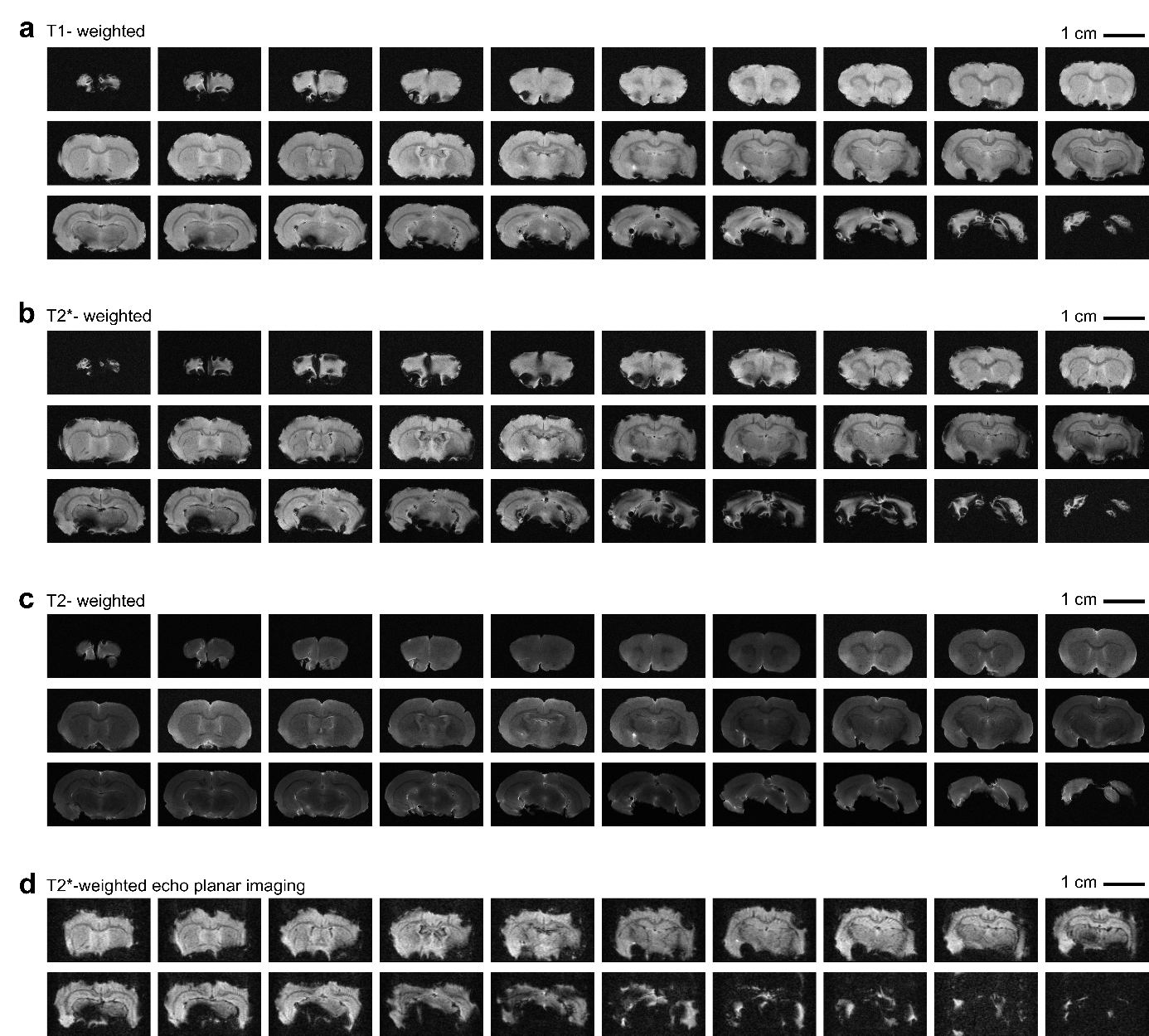


**Figure S14.** MRI images of ex vivo rat brain with B-Sensor.


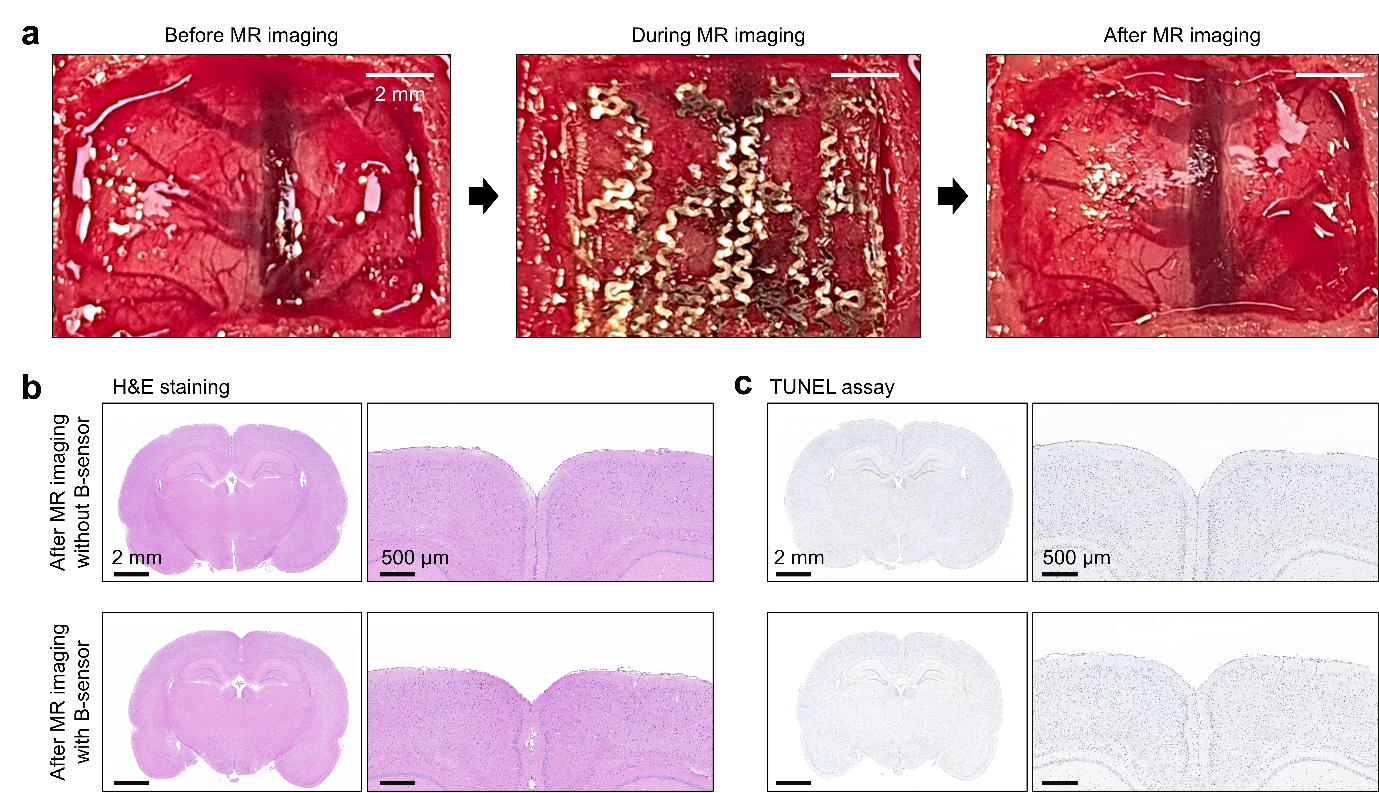


**Figure S15. Evaluation of MRI-induced tissue heating in vivo.** a) Optical image of the rat brain surface before MRI scanning, during MRI scanning, and after MRI scanning. b) Representative H&E stained images obtained from brain tissues of animals subjected to MRI scanning without B-sensor implantation and after MRI scanning with the B-sensor implanted. c) Representative TUNEL stained images obtained from brain tissues of animals subjected to MRI scanning without B-sensor implantation and from animals after MRI scanning with the B-sensor implanted. Brain tissues for histological analysis (b–c) were collected 3 h after MRI scanning.

| **MR safety** | **Degradability** | **Conformability** | **Adhesion** | **Shape morphability** | **Stretchability** | **Substrate** | **Electrode material** | **Reference** |
| --- | --- | --- | --- | --- | --- | --- | --- | --- |
| Yes | Yes | High | Yes | Yes | Stretchable | Biodegradable self-healing polymer | Mo | This work |
| Not reported | No | Low | No | No | Flexible | PI | Pt | [1] |
| Not reported | No | High | Yes | No | Flexible | Silk fibroin / patterned polyimide | Au | [2] |
| Not reported | No | Low | No | No | Flexible | PI | Pt | [3] |
| Yes | No | Moderate | No | No | Stretchable | PDMS | Pt NP / PDMS | [4] |
| Not reported | No | High | No | No | Stretchable | Polyvinyl alcohol | PEDOT:PSS | [5] |
| Not reported | No | High | Yes | Yes | Stretchable | Self-healing polymer / patterned PI | Au | [6] |
| Not reported | Yes | Low | No | No | Flexible | PLCL-PLGA | Mo / Mg | [7] |
| Not reported | Yes | Low | No | No | Flexible | PLGA | Mo / Si | [8] |
| Not reported | Yes | Low | No | No | Flexible | PLGA | Mo / Si | [9] |
| Not reported | Yes | Low | No | No | Flexible | PLGA | PEDOT:PSS / Au | [10] |

Table R1. **Comparison of representative flexible and stretchable ECoG platforms.** Mo, molybdenum; Pt, platinum; Mg, magnesium; Si, silicon; Au, gold; PEDOT:PSS, poly(3,4-ethylenedioxythiophene):poly(styrenesulfonate); PI, polyimide; PDMS, polydimethylsiloxane; PLCL-PLGA, poly(L-lactide-co-ε-caprolactone)/poly(lactic-co-glycolic acid); PLGA, poly(lactic-co-glycolic acid).

**References**

1. J. Viventi, D.-H. Kim, L. Vigeland, *et al.,* "Flexible, foldable, actively multiplexed, high-density electrode array for mapping brain activity in vivo." *Nature Neuroscience* 21 (2011): 1599-1605.

https://doi.org/10.1038/nn.2973

2. D.-H. Kim, J. Viventi, J. J. Amsden, *et al.,* "Dissolvable films of silk fibroin for ultrathin conformal bio-integrated electronics." *Nature Materals* 9 (2010): 511–517.

https://doi.org/10.1038/nmat2745

3. B. Rubehn, C. Bosman, R. Oostenveld, P. Fries, and T. Stieglitz, "A MEMS-based flexible multichannel ECoG-electrode array." *Journal of Neural Engineering* 6, no. 3 (2009): 036003.

https://doi.org/10.1088/1741-2560/6/3/036003

4. F. Fallegger, G. Schiavone, E. Pirondini, *et al.,* "MRI-Compatible and Conformal Electrocorticography Grids for Translational Research." *Advanced Science* 8, no. 9 (2021): 2003761.

https://doi.org/10.1002/advs.202003761

5. S. Oribe, S. Yoshida, S. Kusama, *et al.,* "Hydrogel-Based Organic Subdural Electrode with High Conformability to Brain Surface." *Scientific Reports* 9, no. 1 (2019): 13379.

https://doi.org/10.1038/s41598-019-49772-z

6. S. Lee, J. Kum, S. Kim, *et al.,* "A shape-morphing cortex-adhesive sensor for closed-loop transcranial ultrasound neurostimulation." *Nature Electronics* 7, no.9 (2024): 800-814.

https://doi.org/10.1038/s41928-024-01240-x

7. J.-Y. Bae, G.-S. Hwang, Y.-S. Kim, *et al.,* "A biodegradable and self-deployable electronic tent electrode for brain cortex interfacing." *Nature Electronics* 7 (2024): 815–828.

https://doi.org/10.1038/s41928-024-01216-x

8. K. J. Yu, D. Kuzum, S.-W. Hwang, *et al.,* "Bioresorbable silicon electronics for transient spatiotemporal mapping of electrical activity from the cerebral cortex." *Nature Materials* 15 (2016): 782–791.

https://doi.org/10.1038/nmat4624

9. M. Cho, J.-K. Han, J. Suh, *et al.,* "Fully bioresorbable hybrid opto-electronic neural implant system for simultaneous electrophysiological recording and optogenetic stimulation." *Nature Communications* 15 (2024): 2000.

https://doi.org/10.1038/s41467-024-45803-0

10. M. Wu, K. Yao, N. Huang, *et al.,* "Ultrathin, Soft, Bioresorbable Organic Electrochemical Transistors for Transient Spatiotemporal Mapping of Brain Activity" *Advanced Science* 10, no. 14 (2023): 2300504.

https://doi.org/10.1002/advs.202300504

**Supplementary Movies**

**Movie S1.** Tissue adhesion of Alg-CA coated PPU

**Movie S2.** Spontaneous shape morphing property of Alg-CA coated PPU

**Movie S3.** Spatiotemporal mapping of seizure with B-Sensor
